# neuroAIx-Framework: design of future neuroscience simulation systems exhibiting execution of the cortical microcircuit model 20× faster than biological real-time

**DOI:** 10.3389/fncom.2023.1144143

**Published:** 2023-04-20

**Authors:** Kevin Kauth, Tim Stadtmann, Vida Sobhani, Tobias Gemmeke

**Affiliations:** Chair of Integrated Digital Systems and Circuit Design, RWTH Aachen University, Aachen, Germany

**Keywords:** neuromorphic computing architectures, FPGA cluster, cortical microcircuit, spiking neural networks (SNN), computational neuroscience, parallel computing, rapid prototyping, accelerated simulation

## Abstract

**Introduction:**

Research in the field of computational neuroscience relies on highly capable simulation platforms. With real-time capabilities surpassed for established models like the cortical microcircuit, it is time to conceive next-generation systems: neuroscience simulators providing significant acceleration, even for larger networks with natural density, biologically plausible multi-compartment models and the modeling of long-term and structural plasticity.

**Methods:**

Stressing the need for agility to adapt to new concepts or findings in the domain of neuroscience, we have developed the neuroAIx-Framework consisting of an empirical modeling tool, a virtual prototype, and a cluster of FPGA boards. This framework is designed to support and accelerate the continuous development of such platforms driven by new insights in neuroscience.

**Results:**

Based on design space explorations using this framework, we devised and realized an FPGA cluster consisting of 35 NetFPGA SUME boards.

**Discussion:**

This system functions as an evaluation platform for our framework. At the same time, it resulted in a fully deterministic neuroscience simulation system surpassing the state of the art in both performance and energy efficiency. It is capable of simulating the microcircuit with 20× acceleration compared to biological real-time and achieves an energy efficiency of 48nJ per synaptic event.

## 1. Introduction

Computational neuroscience is a very broad and multi-faceted research field. Starting at the molecular level up to the modeling of human behavior, a very wide scale in time and space is spanned with no technical system capable of simulating the complete stack. While tremendous progress has been made in recent years by the community, the question of how the brain transforms information is still a puzzle. To gain deeper insights, the simulation of neuronal network models of natural density is considered essential.

Today, there exist various neuroscience simulators targeting different resolutions and abstraction levels. Examples include dedicated neuromorphic hardware systems such as SpiNNaker (Furber et al., 2013), BrainScaleS (Schemmel et al., 2008), Bluehive (Moore et al., 2012) or Loihi (Davies et al., 2018), and software frameworks like NEST (Gewaltig and Diesmann, 2007) running on conventional CPU-based systems or GeNN (Yavuz et al., 2016) and NeuronGPU (Golosio et al., 2021) running on GPU-based systems. Recent iterations of these systems target a broader range of tasks for example in the area of Machine Learning, and feature higher degrees of flexibility and efficiency (Mayr et al., 2019; Billaudelle et al., 2020). In addition, recent dedicated systems exist that target the simulation at higher degrees of abstraction (Wang et al., 2018) or aim at solving Machine Learning tasks (Panchapakesan et al., 2022). On one side, the mentioned variety together with advances in computational capabilities and the development of simulator-independent model description languages (e.g., PyNN by Davison et al., 2009) pushed the domain of computational neuroscience to study neural network models of increasing complexity. These include large-scale models such as the cortical mesocircuit (Senk et al., 2018) and multi-area model (van Albada et al., 2020). On the other side, spiking networks are gaining traction in industry to address technical problems as targeted by the Loihi platform (Dey and Dimitrov, 2021).

Among the various neuroscience models, the cortical microcircuit (Potjans and Diesmann, 2014) has become a widely-used benchmark to evaluate simulators (Knight and Nowotny, 2018; van Albada et al., 2018; Rhodes et al., 2019; Knight et al., 2021; Heittmann et al., 2022; Kurth et al., 2022), driving novel systems toward innovative designs and higher performance. For instance, an initial mapping on SpiNNaker was operating 20× slower than biological real-time (van Albada et al., 2018). However, deeper understanding including insights into the learning processes of the human brain requires the simulation of long-term neurodynamical processes, i.e., simulations at higher speed. Along this line, solutions based on GPU-enhanced simulators reduced the slow-down to 1.8 × (here and in the following: with regards to biological real-time) (Knight and Nowotny, 2018). Shortly thereafter, the first real-time simulation of the cortical microcircuit was run on SpiNNaker (Rhodes et al., 2019). To the best of our knowledge, the fastest realization uses the IBM Neural Supercomputer achieving an acceleration of 4.06 × (Heittmann et al., 2022).

The major challenge in designing such a system lies within the required flexibility to accommodate new insights from the neuroscience domain that change the specification and requirements. Some years back, simulation time was commonly progressing in discrete steps of 1 ms whereas nowadays, 0.1 ms are used to better capture the short delays along local axons (Potjans and Diesmann, 2014). Similarly, a supported number of 1,000 synapses per neuron was assumed to be sufficient for many earlier systems. Recent insights suggest the average fan-out to be higher, causing major performance losses on these systems. The required degree of biological realism is still under discussion including seemingly simple questions such as required numeric precision or suitable compartment size of dendrites. The resulting volatility in biological models must be taken into account in the design of new systems. More complex questions relate to the modeling of plasticity. Nowadays, three-factor rules (Kuśmierz et al., 2017) that modulate simple spike-timing-based learning mechanisms are considered. These advances pose new requirements on the computation and communication capabilities. To account for this and other developments, computational neuroscience requires a next-generation system to help to efficiently gain new insights. In turn, these insights will redefine the technical specification for this system. This *chicken-and-egg* problem is best addressed in an evolutionary process relying on reciprocal advances on both sides.

In this ever-changing environment, we derive the need for a *flexible* system, capable of performing simulations of large-scale neuronal networks *observable* down to membrane potentials. Its complexity has to reach realistic densities, simulated in an *accelerated* fashion to also capture long-term effects, e.g., regarding synaptic plasticity. Furthermore, the system behavior needs to be fully *deterministic* in order to reproduce results, operate with intermittent system states and precisely capture the impact of parameter variations in the neuroscience model (as opposed to observing irrelevant variations relating to the simulation environment). Finally, we target a *scalable* system that is future-proof toward more complex or larger models. To achieve appropriate speed-up at the same time, the problem needs to be distributed over many compute nodes to overcome computation and routing bottlenecks, among others.

**Figure 1:**
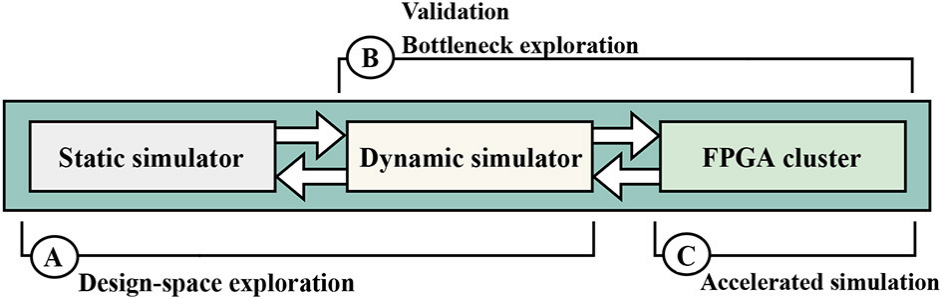
The proposed development framework for accelerated neuroscience simulations. **(A)** The static and dynamic simulators are jointly used for exploring the design space. We calibrate the dynamic simulator according to the performance analysis done by the static simulator and then update our analytical model considering the observed dynamic behaviors. **(B)** The dynamic model will be validated and calibrated by the FPGA cluster. Afterward, the bottlenecks of the design can be studied by both simulation platforms available here. **(C)** The FPGA cluster is used for both validating the dynamic simulation and fine-tuning its parameters. For this, it can perform accelerated simulations of biological neural networks.

In this work, we present our *neuroAIx-Framework* which is suited to evaluate and benchmark prospective system architectures in a highly flexible and performant manner. It consists of three pillars as illustrated in Figure 1—(1) an empirical modeling tool (*static simulator*) for fast design space exploration at a coarse resolution, (2) a virtual prototyping platform (*dynamic simulator*) for accurate performance estimations, and (3) a cluster of interconnected FPGA boards (*FPGA cluster*) for evaluation and simulator calibration.

In the following sections, the workflow using this framework is illustrated with various examples. In particular, we focus on steps B and C by realizing the FPGA cluster which should not be considered a fixed, final solution to the quest of finding the future neuroscience simulation platform. Instead, the cluster is an evaluation platform that functions as a proof-of-concept for this workflow. We use it to refine and calibrate the first two pillars such that they provide more trustworthy quantitative results in future explorations. While the third pillar is mainly intended as an emulator of a future system, it is fully functional and already speeds up neuroscience simulations. More specifically, we are able to simulate the cortical microcircuit model at an acceleration of 20× which is, to the best of our knowledge, the fastest solution so far.

The proposed framework can be utilized to constrain the design space of neuroscience simulation systems, identify the costs and bottlenecks, explore solutions and validate ideas. In our previous work, we studied suitable communication architectures—a major bottleneck in accelerated simulation of large-scale networks—utilizing the static and dynamic simulators (Kauth et al., 2020). Similarly, Kleijnen et al. (2022) focused on simulation regarding heterogeneous neural networks and corresponding mapping algorithms. However, this work extends this to the characterization of all relevant building blocks that are necessary for a dedicated neuroscience simulation system, and sketches their implementation in the presented evaluation platform. We believe that the fast-prototyping feature of our method is an essential aspect to close the gap between system design and the fast-moving domain of computational neuroscience, leading to even faster progress in both domains in the future.

To summarize, this paper presents the realization of a coherent framework to explore future neuroscience simulation systems. It allows to

1. Perform fast system exploration and precisely analyze requirements of larger scale models emulating system behavior in a cycle-accurate fashion,
2. Simulate the 1 mm^2^ cortical microcircuit model at an acceleration of more than 20×, and
3. Support computational neuroscience research, aiding the evaluation of new neuron models, novel plasticity rules as well as parameter sweeps in an accelerated fashion.

This contribution intends to establish a basis for future interaction between the neuroscience community and engineers working toward next-generation large-scale neuromorphic accelerators.

## 2. Materials and methods

### 2.1. Static simulator

Expressing a platform's performance as function of model and system parameters in an analytical form leads to overly complex equations, especially considering the stochastic instantiation of synapses or spikes. As our goal is to build a flexible and, importantly, scalable platform, this pillar focuses on guiding the design of a suitable communication architecture. Hence, we developed a *C++*-based numerical simulator to extrapolate system performance in a highly efficient manner. It is used to explore communication architectures, not yet accounting for other bottlenecks such as memory and computation.

The simulation tool operates on the assumption of a homogeneous network topology. One arbitrary node is used as starting point in the calculations. According to a well-defined average spike rate or based on the evaluation of some existing simulation, the selected nodes emit spikes with a specific probability. These spikes travel to a randomly distributed set of target neurons. Based on an evaluation of the distance each packet has to traverse, a numerical solver calculates bandwidth requirements and speed-up as bounded by the communication. This empirical approach has been cross-validated with examples that are simple enough to be expressed in an analytical form. As one example, the bandwidth requirement in a broadcast approach for a mesh-like network topology is driven by the number of neurons per node, the firing rate, the system's acceleration factor, the number of target nodes and the size of each spike message (Kauth et al., 2020).

The fast collection of quantitative data using this approach enables quick architectural exploration and early pruning of unsuitable directions. For example, the required number of network hops to deliver a package severely constrains the choices of suitable network topologies and routing schemes. The modeling of the most promising candidates can be refined while larger sample sets increase the confidence in the numeric results. This approach considers average system loads only, i.e. there is no notion of queuing, no unbalanced distribution of tasks nor any other dynamics considered in the evaluation of the system. Hence, we call this pillar *static simulator*. While it provides a much simplified assessment of the capabilities of different communication architectures, its key benefit is its speed—networks from thousands to millions of compute nodes can be evaluated in seconds, enabling the exploration of a vast design space.

### 2.2. Dynamic simulator

As many system architecture iterations will be necessary due to the mentioned chicken-and-egg problem, rapid prototyping is essential. Hence, it is necessary to virtually evaluate the prospective system architectures before finalizing the specification or even starting concrete design activities. For this, we developed a generic virtual prototype modeling architectural components like memories, routers and schedulers at varying levels of accuracy. These range from coarse behavioral models down to bit and cycle-true functional descriptions. It is an event-driven simulation model that emulates hardware platforms to capture their dynamic behaviors. In contrast to the static simulator, this *dynamic simulator* incorporates dynamic behavior such as congestion, and not only focuses on communication aspects but also on memory and computation.

The dynamic simulator is written in SystemC, a *C++* library used to model functional aspects of hardware systems with a high-level software language. The architectural components of the hardware are encapsulated in corresponding *modules*—SystemC's basic building blocks—connected to each other using *ports*. In a bottom-up perspective, the core module is the compute node that updates the state variables during each timestep. It aggregates all computations related to the neurons hosted on a specific node in the system. Details of the actual computation are omitted. Instead, only the computation latency is used to capture performance capabilities as function of neuronal model complexity. For this, the module absorbs spikes and generates new ones according to a predefined statistical distribution. Furthermore, each node runs its own synchronization process which is necessary for cycle-accurate behavior. Details on the synchronization process will be elaborated in Section 2.3.1.5.

In the mid-level, the network module has been designed to connect the instantiated nodes according to the specified topology by cable modules. These cables are implemented as SystemC *channels*. Relevant system properties such as transmission delay and bandwidth are captured by specifying the cable length, transceiver delay and bandwidth.

The top-level module covers the user interface, starts the simulation, sets the configuration and calculates statistics at the end of the simulation. Design parameters can be specified in a configuration file at run-time, organized in four categories: biological parameters (e.g., firing rate, number of neurons, connectivity distribution), simulation parameters (e.g., number of simulation steps), interconnect (e.g., topology, routing, bandwidth) and hardware architecture parameters (e.g., number of neurons per node, number of workers, buffer depths, pipeline stages).

The modular and hierarchical design of the dynamic simulator allows to test varying scenarios, develop different network topologies or even communication schemes without changing the aforementioned modules, as it already contains all necessary building blocks. Results of dynamic simulation are fed back to the static simulator to improve accuracy by refining its empirical models. At the same time, the dynamic simulator itself can be refined using measurement results from the physical system.

### 2.3. FPGA cluster

As key component, the FPGA cluster is a fully operational platform capable of running large-scale neural network simulations. At the same time, the framework incorporates a high degree of flexibility to evaluate alternative design choices. On one side, these are triggered by the exploration and profiling within the different pillars. On the other side, they emanate from new learnings in neuroscience and the respective research in modeling biological processes of the brain. Our objective is to conceive and realize a platform that provides both the necessary flexibility and adequate performance to evaluate meaningful test cases. In the following, we will therefore elaborate general design principles and hardware concepts needed to implement such a flexible platform. For each component, we then sketch certain design decisions employed in our evaluation system. Measurement results on this system are used later to calibrate the dynamic and static simulators, which then in turn allow for more realistic design space explorations.

**Figure 2:**
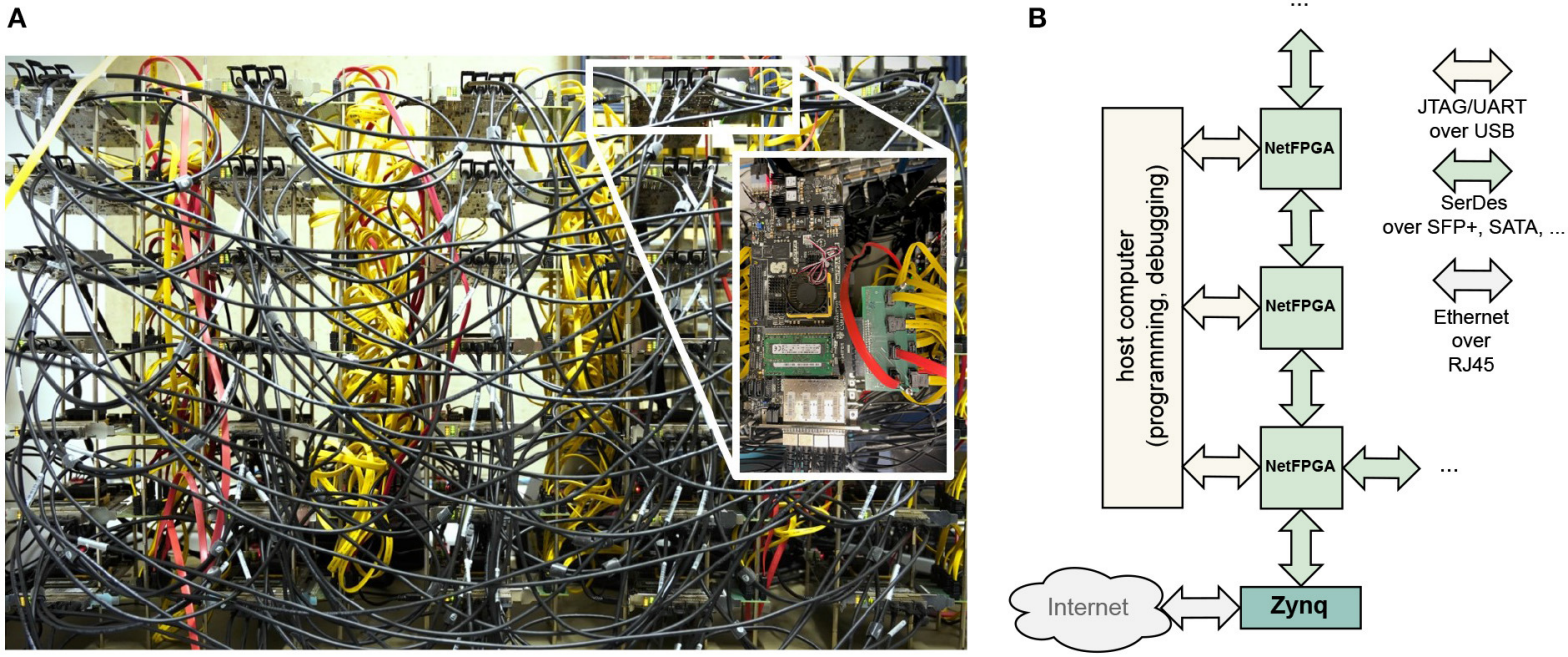
**(A)** Lab setup of physical cluster of 7 × 5 *NetFPGA SUME* boards. The boards are horizontally connected via black SFP+ cables and vertically connected via red and yellow SATA 6G cables. The exact network topology will be discussed in chapter 3. **(B)** Protocol and physical links used in node-to-node and node-to-host communication.

To this end, we designed a cluster of 35 FPGA boards. FPGAs (FPGAs), in contrast to dedicated ASICs (ASICs), allow for rapid prototyping, while at the same time offering a wider flexibility and potential performance than GPU-based implementations. As the basis of this cluster we chose the NetFPGA SUME board (Zilberman et al., 2014) as it provides a high number of transceivers with a theoretical total bandwidth of over 100Gbps and two memory channels to the 8GB DDR3 with direct connections to the programmable logic. In the current setup, 4 SFP+ ports with each 6.25Gbps and 10 SATA ports with each 6Gbps are used for interconnecting the FPGA boards and host communication. Eight of these SATA ports are made available using a custom PCIe breakout board. Although not all of these connections are required for the 35 node setup, they are well suited for evaluating different network topologies. For an even larger number of nodes, reconfigurable switching solutions are recommended, see e.g., Meyer et al. (2022). Figure 2A shows a picture of the connected cluster.

In any case, the developed environment is not dependent on any specific FPGA board, number of nodes, port count or network topology. This flexibility is considered a key advantage over fixed neuromorphic simulators in the context of exploring future hardware architectures. Apparently, the added flexibility leaves room for further improvements in performance once a suitable architecture has been identified.

#### 2.3.1. Node architecture

**Figure 3:**
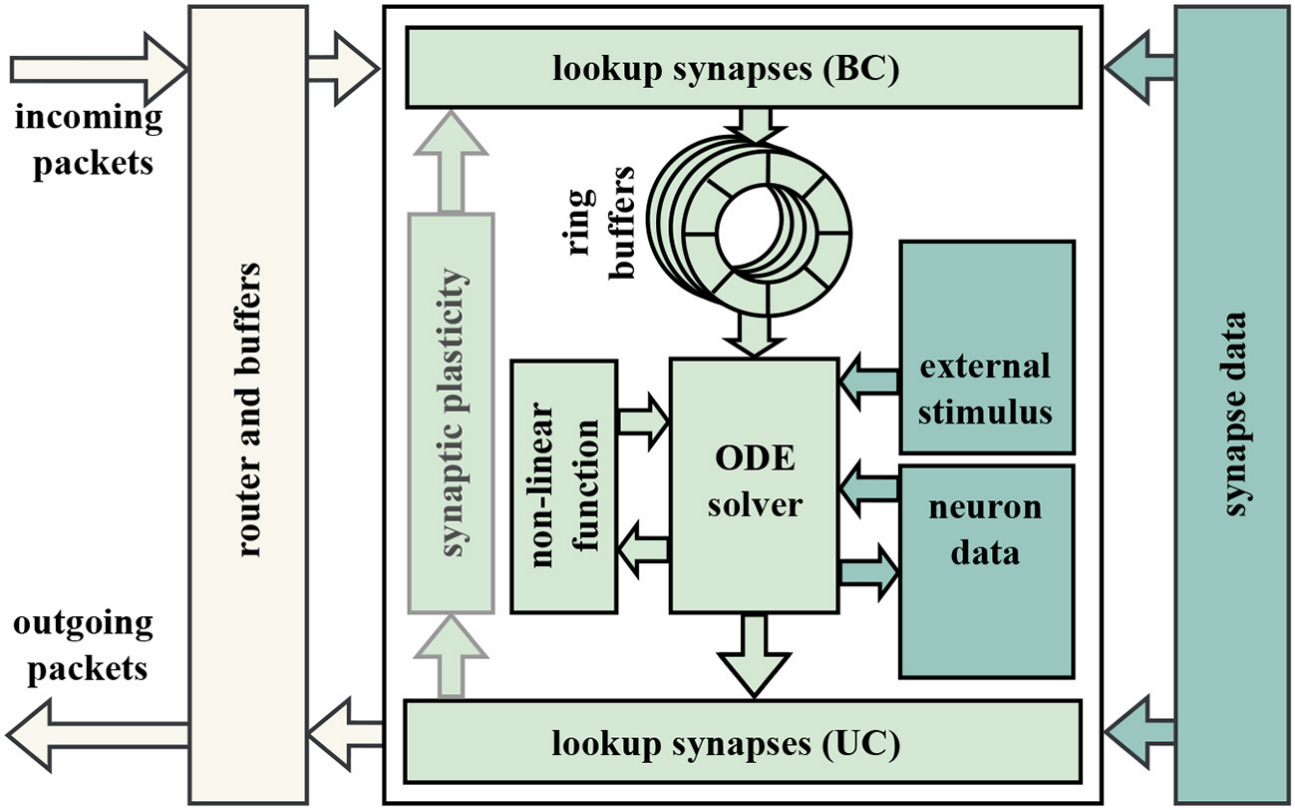
Schematic illustrating the node of a generic distributed system for the simulation of biological neural networks. The left part represents the communication interface of a node to the network. The center part depicts the internal structure of a node. Synaptic data commonly resides in an external off-chip memory (right part).

Due to the homogeneous network architecture of our system, the neuron mapping to the individual nodes is irrelevant for neural networks with roughly evenly-distributed connectivity. As this is considered realistic (Potjans and Diesmann, 2014), our current implementation distributes all neurons equally in a round-robin fashion. The individual nodes handle all necessary processing related to the local neurons and communication of spikes. The simulation is time-driven (as opposed to event-driven)—each neuron's state variables are updated in successive discrete timesteps, often 0.1 ms, set by the minimal synaptic latency (Brette et al., 2007). In the following sub-section, we present the components of a neuromorphic accelerator architecture. A high-level overview is depicted in Figure 3.

##### 2.3.1.1. Workers

The actual computation of the neuronal dynamics is scheduled to the available workers at the node. Firstly, this computation requires a fast memory for the state variables of each neuron available at the worker. We opted for on-chip BRAM—blocks of SRAM—since the required capacity is low while BRAMs (BRAMs) offer a single-cycle access latency. Secondly, the worker contains the implementation of a neuron model. This either requires the computation of a simple matrix multiplication for analytically-solvable models such as LIF neurons with CUBA synapses (Rotter and Diesmann, 1999) or entails the execution of a numeric ODE solver to determine the solution for more complex neuron models such as Izhikevich (Izhikevich, 2003). As the neuron model processing can be executed in parallel, increasing the number of workers provides direct speed-up here. The interface to these workers and their memories requires only input and output streams of spikes, making the neuron model easily interchangeable.

The state update of a neuron can result in the creation of an action potential. In that case, a corresponding network message is created and forwarded to a router. Apparently, the router also passes incoming spikes to the workers. Depending on the time of origin and the synaptic delay, incoming spike messages are to be considered in the computation in a specific timestep. Since this timestep does not necessarily correspond to the current one, spike messages have to be stored temporarily. The impact of multiple spikes onto the same target neuron in the same timestep can typically be lumped into one synaptic input (Rotter and Diesmann, 1999). This offers the possibility of not having to buffer an arbitrarily large number of incoming messages. Instead, only a single synaptic input for each neuron and any future timestep has to be stored. Since the number of future timesteps which can contain such synaptic inputs is limited by the largest synaptic delay present in the neural network, it is usually implemented as a ring buffer. Synaptic inputs are forwarded by the purely combinational local router to the target ring buffers in a round-robin fashion. In turn, the worker reads the synaptic inputs to all neurons for the current timestep sequentially from the ring buffer. As new spikes can be captured in the ring buffer asynchronously at any time, the ring buffer and the worker are decoupled in terms of congestion.

In our system, each worker can calculate up to 255 LIF neurons with CUBA synapses, with 10 of these workers being instantiated per FPGA. The number of neurons is largely limited by the available BRAM which is used for the ring buffers. Thereby, models with up to 89,600 neurons can be handled by the cluster of 35 FPGAs. While more workers, and hence neurons, could potentially fit onto the FPGA with further optimization, this is already sufficient for simulating the cortical microcircuit model, and spending effort on a highly specific and optimized realization is not in the focus of the exploration. Running at a frequency of 189.383 MHz (chosen to be synchronous to the DRAM memory to minimize clock domain crossing) and 30 pipeline stages, the neuron dynamic computation of a fully occupied node takes around $\frac{255+30}{189.383\text{MHz}}\approx 1.5\mu$s. In this configuration, the corresponding ring buffers can store incoming spikes of up to 64 future timesteps for excitatory and 32 future timesteps for inhibitory synapses, as determined by the maximum synaptic delays in the cortical microcircuit model.

##### 2.3.1.2. Synapse lookup

Each neuron can be connected to many thousands of other neurons. Hence, each node requires a mechanism to assign spikes of presynaptic to postsynaptic neurons. This assignment is stored in the form of so-called synaptic lists and contains information about the assignment by means of unique neuron identifiers as well as the weight and delay of all related synapses. Due to the large number of synapses in natural-density neural networks, these lists must typically be stored in an off-chip memory such as a DRAM. The point in the system where these lists are accessed (the *lookup*) depends on the used casting scheme. If the network operates in broadcast mode, the presynaptic neuron ID of each generated spike is simply distributed over the entire network. The receiving nodes then lookup the synaptic information (weights, delays, targets) for all synapses between the presynaptic neuron and all local postsynaptic neurons for every incoming spike message. On the other hand, when using unicast the lookup is performed directly on the spike-emitting worker, prior to sending the spike message to the outgoing router. In this scheme, all postsynaptic neurons are addressed individually with a unicast message. In a system with flexibility to support both casting schemes, the hardware performing this lookup should be added both before and behind the workers and then be bypassed depending on the used casting scheme, as shown in Figure 3. Our lookup module was designed with this flexibility in mind, as it supports varying the number of prefetched words and parallel memory accesses at run-time. This allows us to tune it based on bottleneck measurements and simulation results from the dynamic simulator.

##### 2.3.1.3. Router

In the present design, each FPGA node contains a dedicated router unit. Given the application requirements, our router is designed for ultra-low latency communications and high bandwidth of more than 12 Gbit/s per port while all ports can operate fully in parallel. Since the cluster is a development platform, it is also essential to be topology-agnostic, e.g., be flexible in terms of number of inputs and outputs ports. Furthermore, our router currently supports three different modes: emulation, bottleneck measurements and debugging. The last two modes follow simple routing algorithms, for example, forwarding packets in a certain direction. The emulation mode supports both unicasting and broadcasting, a variety of routing algorithms [e.g., best neighbor, windmill and xy routing (Kauth et al., 2020)], and various mesh- and tree-based topologies. Based on the packet type (synchronization packets, configuration packets or spike packets), the router redirects packets to the correct target(s). In all cases, a prioritized round-robin arbiter takes care of time-critical messages first.

##### 2.3.1.4. Memory

A significant amount of data has to be sent to and received from such a simulation platform, comprising configuration data, dynamic neuron state information and result data (spikes, voltage traces, etc.). The on-chip BRAM is typically insufficient to hold all of this. Larger off-chip storage (e.g., DRAM) offers plenty of storage capacity but introduces potential throughput and latency bottlenecks. In the present realization, the static and dynamic state information of the neuron models is kept in the BRAM providing a reasonable trade-off between performance and storage requirement. With typically less than 1kB needed for each neuron's state and ring buffer, the available 6.77MB BRAM on our system can already accomodate many thousands of neurons. The memory requirement for the connectome, however, is considerably higher. In the case of the microcircuit with about 300 million synapses, several gigabytes of data are anticipated. Although this amount of data is distributed across all nodes, the sparse adjacencies require a sophisticated form of organization. A simple approach is to store the data in a single connection list per neuron. Since these lists have different lengths, base addresses must additionally be stored in the form of a lookup table. An alternative way is to pad the lists up to a common length. Although this method wastes some memory, it reduces latency by avoiding a second non-linear memory access.

In our case, each synapse consists of a 16bit target neuron identifier, an 8bit delay, and a 32 bit fixed-point weight, resulting in 2.1GB for the microcircuit connectome. This connectome would already fit into the DRAM of a single node, while it would not even come close to fit into the BRAM of the complete cluster. Since we are targeting a distributed system, the connectome is split over all nodes and stored as padded synaptic lists with common length. On the one hand, this padding causes synaptic lists to occupy 200 MB of DRAM memory on each node. On the other hand, the memory address of any synaptic list can be calculated (using source neuron identifier and fixed list length). On every incoming spike, a DRAM read access is started to retrieve the corresponding synaptic data. The amount of data requested depends on the neuronal fan-out.

Since the use of external memory not only limits the size of the synaptic lists, but also drastically restricts the achievable simulation speed due to bandwidth limitation and comparatively high latency, any approach completely eliminating time-critical external memory accesses would be preferable. For instance, algebraic definitions of the connectivity pattern would allow to compute synapse configuration data on-the-fly, which reduces memory requirements significantly (Roth et al., 1995). When combined with online computation of other parameters, such as synaptic efficacy or axonal delay, based on deterministic random distributions, external memory is no longer a critical resource (Wang et al., 2014). However, as this precludes the implementation of neuronal plasticity (where individual weights will have to be adjusted and new synapses added) and the simulation of specific connectomes (e.g., extracted from actual biological tissue) we excluded this method in the current exploration.

##### 2.3.1.5. Synchronization

Due to fluctuating loads, caused by local spike bursts or coincidentally increased routing via certain nodes, the individual nodes of the network can reach locally varying processing latencies. A commonly used scheme to prevent the nodes from drifting apart are global barrier messages (e.g. Heittmann et al., 2022). Global synchronization, however, causes the entire system to run at the speed of the slowest node. Therefore, we use local synchronization in our system. Instead of forcing all nodes in the network to the same timestep, we limit the maximum time difference of directly neighboring nodes to the minimum synaptic delay across all synapses. This enables compensation of fluctuations in the simulation speed, maximizing the overall speed of the system.

**Figure 4:**
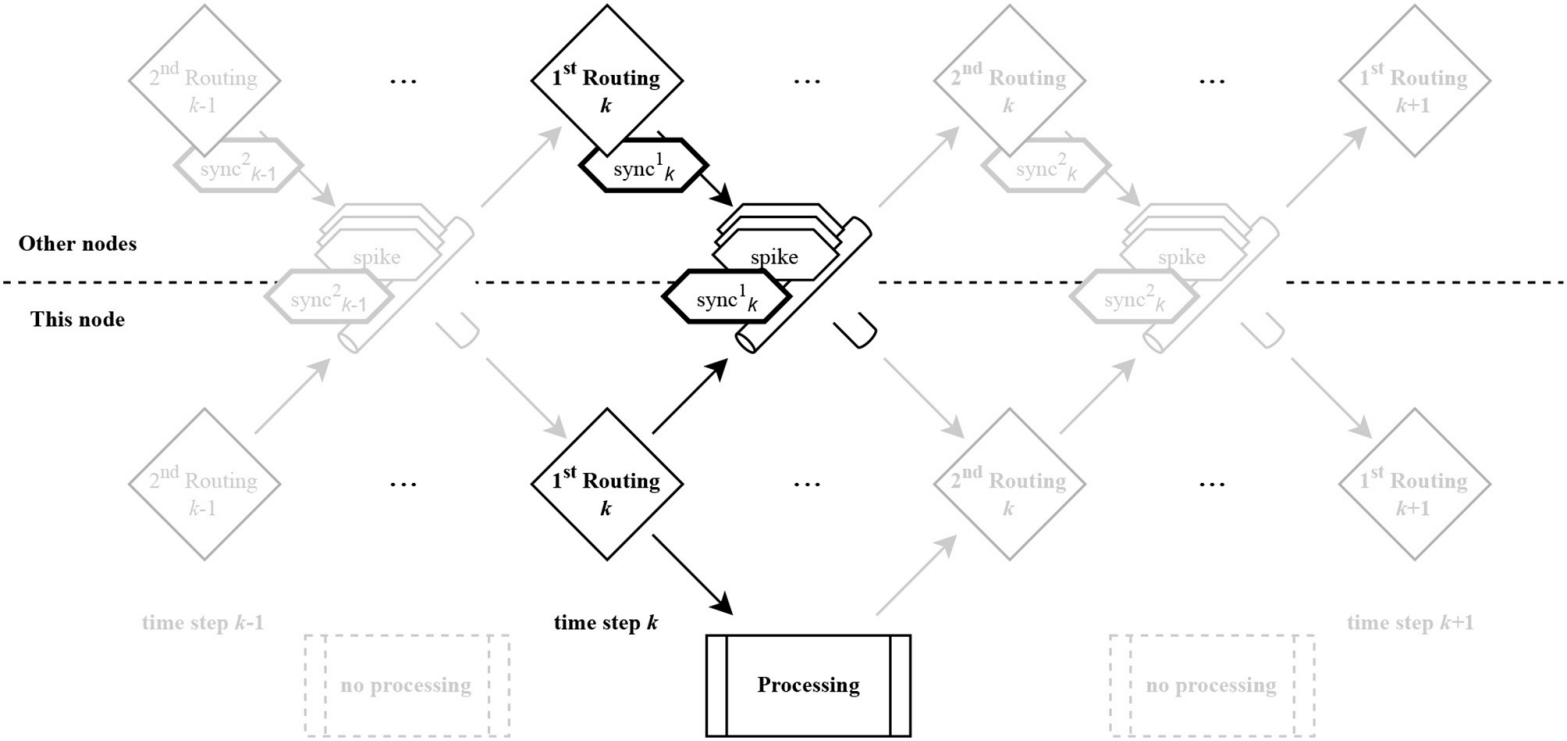
Synchronization across nodes using two neighbor-to-neighbor synchronization events per timestep.

In our synchronization scheme, a synchronization message is sent to each neighbor after each computation of the neuronal dynamics (see Figure 4). This message is always sent after the last generated spike for the corresponding direction. When a node receives such a message, it can assume that it has received all spikes for this timestep from the corresponding neighbor. Since a computation is started only after all synchronization messages have been received and either forwarded or consumed, it can be guaranteed that each spike message has been forwarded by at least one node. However, the topology we use has a worst case latency of two hops (topology and routing algorithm will be derived and explained in Section 3), while the microcircuit has some synapses with a delay of only one timestep. Therefore, a second synchronization per timestep is used, which, however, is not initiated after completion of the computation, but after receiving the first synchronization message of all neighbors. Accordingly, the next computation is only started after all second synchronization messages have been received.

In general, the number of synchronizations per timestep can be set to the worst case number of network hops required by a message, as each synchronization guarantees that a message will travel at least one hop per synchronization step. This procedure is sufficient for timely arrival of all spikes but not always necessary depending on network bandwidth and load. Performing multiple synchronizations increases the latency between timesteps and consequently slows down the simulation. In our implementation, this can be avoided by manually selecting a smaller number of synchronizations and monitoring spikes arriving too late via corresponding error registers. This highlights the trade-off between correctness and reproducibility on the one hand, and speed-up on the other.

#### 2.3.2. Interconnect

The design of an efficient interconnect architecture both in between nodes and from nodes to host systems is crucial. While the former can limit weak and strong scalability, the latter is generally considered a challenging task for neuromorphic platforms where large amounts of data need to be transferred from and back to host systems regularly (Knight and Nowotny, 2018) causing the setup time to exceed simulation time in many systems (Schemmel et al., 2008; Furber et al., 2013). For both challenges, wireless links seem like a promising solution, being able to deliver all spikes in one hop in-between nodes, and sending results back to a number of hosts using the same technology. But while there has been some work on wireless HPC with technologies like free-space optic or 60GHz radio communication (Li et al., 2020), the reduced energy-efficiency and high error rates make this option at the current state infeasible. Suitable alternatives and our respective implementation will be outlined in the following.

##### 2.3.2.1. Node to node

As a deterministic neural network simulation requires in-time arrival of spike messages, it is clear that latency and bandwidth are major factors determining system performance - the less network hops a message needs to arrive at all destinations, the better (Kauth et al., 2020). Communication via SerDes MGT, coupled with 64/66b encoding, offer a good tradeoff between low latency, high-speed and reliability, given a suitable transceiver technology. However, the scalability of the communication architecture is limited by the topology, routing algorithms and casting methods used in the system. In our previous work, we have assessed conventional electrical/optical toroidal mesh topologies and showed how such connection schemes are favorable for neighbor-only communication (Kauth et al., 2020). We also demonstrated that they fail transferring spikes to non-neighbor nodes in an accelerated fashion due to excessive latency, especially when white matter connections are simulated. To tackle this, we introduced so-called *long hop* connections—superimposed meshes—and appropriate routing algorithms. The exact set of communication methods employed in our system will be derived from the dynamic simulator in Section 3. As mentioned before, the presented FPGA cluster builds on dedicated SerDes transceivers as available in modern off-the-shelf FPGAs.

As communication errors are inevitable, proper error handling is essential. While biological neural networks are inherently noisy (Rolls and Deco, 2010), the exact type and amplitude of this noise must remain a *model* parameter. In a deterministic system, we need to be able to adjust the noise and turn it off at will, which is not possible in the case of uncorrected communication errors. Given the low bit error rate of state-of-the-art communication lines, an acknowledgment-based flow control combining with CRC and re-transmission can guarantee proper error detection and correction. In our exploration platform, we employ an adapted Go-Back-N ARQ algorithm where a received packet is only accepted if CRC checks pass, correct packet order is given *and* receiving buffers are free.

Lastly, deadlock handling is a crucial aspect. The common topology-agnostic deadlock handling scheme—dropping packets in critical cases—is not acceptable in deterministic simulators. In the case of mesh-like topologies, even when containing long hops, a combination of turn restricted routing and virtual channels can avoid deadlocks from happening altogether (Sobhani et al., 2022). In our system, we observed no deadlocks at all in a multitude of large-scale neural network simulations, no matter which topology and routing was used. However, we expect proper deadlock handling to become ever more important in more complex networks.

##### 2.3.2.2. Host to system

A crucial aspect of the targeted exploration platform is the connection from a host machine to the individual nodes. High bandwidths are necessary as neuronal simulations require large amounts of setup and connectivity data to be uploaded. For instance, the cortical microcircuit model (Potjans and Diesmann, 2014) contains around 300 million synapses—with around 8B per synapse (considering synaptic weights, delays and IDs) this results in 2.4GB of data to be uploaded. Most modern FPGAs contain MGTs (MGTs) which are able to handle this amount of data in a few seconds at most. They can for example be interfaced using Gigabit-Ethernet links, providing access to the FPGA cluster over an existing network infrastructure.

However, the employed NetFPGA board does not contain a dedicated TCP/IP stack. To avoid having to use precious FPGA logic for this, we use a dedicated communication node as an interface between the network and the cluster (see Figure 2B). We opted for a Xilinx Zynq board (*ZCU106*) which contains both an ARM processor and FPGA logic. A TCP server running on the ARM sends data received on the RJ45 interface to the FPGA logic via a dedicated AXI bus, and vice versa. The Zynq board is programmed with the same reliability layer and SerDes MGT that are used for communication between the nodes. Thus, no additional logic is required on the simulation nodes.

#### 2.3.3. Configuring and debugging

A system that is used to evaluate different architectures should not only be designed for flexibility, but also be rapidly reconfigurable. Most FPGA boards primarily support configuration using a bitstream, which is transferred via JTAG either directly to the device, or to an on-board flash memory for non-volatile storage. Since this usually requires a direct connection to the board using for example USB, it is not feasible for a large cluster. Depending on whether the FPGA itself has read/write access to the flash memory, two generic configuration schemes for a distributed system are possible. Firstly, if flash access is available, a host machine could directly transfer the bitstream to one connected node, which then broadcasts it through the system. This concept lends itself to our system as broadcast is a necessary feature for any exploration anyway. Secondly, if no flash access is available, partial reconfiguration can be utilized to load an initial design once into the flash. Further modules can be loaded later in the exploration process. However, as our system is still actively developed, we use JTAG and UART over USB for programming and, importantly, debugging individual boards (shown on the left in Figure 2B).

Regardless of the approach chosen, partial reconfiguration can also be used to replace modules that change frequently, such as the neuron model in the workers. This reconfiguration is considerably faster in comparison to complete reconfiguration. In addition, the rest of the system retains its state and can be used directly.

### 2.4. Neural network testcases

#### 2.4.1. Background

The dynamics in biological neuronal networks happen in a wide range in terms of time and space resolution - they are inherently multi-scale (Silver et al., 2007). In the domain of biological neuron models, the LIF model can be considered least complex as it focuses mainly on sub-threshold behavior, while still providing meaningful dynamics in large scale simulations. The existing broad variety of models provides better plausibility (Izhikevich, 2004) with extensions that divide the cell body into multiple compartments.

The rich variety of synapse behavior is reduced in most simulations to the modeling of the PSC. The most basic model assumes the transfer of a charge packet at the time of arrival of a pre-synaptic action potential (delta function). Including some essential temporal behavior the CUBA or COBA models (Vogels and Abbott, 2005) induce an instantaneous rise combined with a more plausible exponential decay.

As much as solving the underlying differential equations of the more complex models will start to impact performance from a certain complexity, it is not part of the subsequent evaluation that focuses on system bottlenecks with respect to handling the spike messages. The regarded large-scale testcases only include LIF neurons with CUBA synapses which can be efficiently solved in a closed form—the so-called *exact exponential integration* (Rotter and Diesmann, 1999).

#### 2.4.2. Microcircuit

The cortical microcircuit model is a full-scale spiking network model of a unit cell of early mammalian sensory cortex, covering 1mm^2^ of its surface. It consists of 77,169 LIF neurons organized into four layers of inhibitory and excitatory populations (Potjans and Diesmann, 2014, Figure 1). The details of the neuron model and its simulation parameters are available in Potjans and Diesmann (2014, Tables 4, 5). The neurons are connected randomly via ~0.3 billion synapses with population-specific connection probabilities. The synaptic strengths as well as transmission delays are distributed normally (Potjans and Diesmann, 2014, Table 5). Besides synaptic connections internal to each neuronal population and in between different populations, every neuron additionally receives Poisson-distributed inputs. These emulate external cortical or thalamic input.

The microcircuit belongs to the smallest networks of natural density, i.e., modeling a realistic number of connections with realistic connection probabilities. At the same time, it exhibits firing rates and irregular activity that match experimental in-vivo findings. Therefore, it poses constraints on communication, computation and memory bandwidth that are both challenging and realistic at this scale. Hence, it has become a well-accepted model by the computational neuroscience community and as a result a benchmark to evaluate neuroscience simulators. While other tasks have been used as benchmarks for individual systems in the past, such as randomly-connected networks of 100k neurons (Stromatias et al., 2013) or variations of the balanced Brunel network (Brunel, 2000), the cortical microcircuit is the most widely adopted and commonly-used benchmark to the best of our knowledge. It will therefore be the basis of later analyses and comparisons. It is important to note that the cortical microcircuit is simulated with a timestep of 0.1ms (as opposed to the 1ms frequently used in the past) to properly account for small synaptic delays of local axons, and a fanout of around 4,000 (instead of 1,000), increasing its complexity compared to older benchmarks [see e.g. the works of Moore et al. (2012) and Furber et al. (2014)].

For the following benchmarks of our system, we use the cortical microcircuit implementation *Potjans_2014* from the PyNEST framework (NEST:: v3.3) without any changes, except setting the *poisson_input* switch to *False* (Eppler et al., 2009). This way, the Poisson input to each neuron is emulated using DC input instead, which was shown by Potjans and Diesmann (2014) to be qualitatively equivalent. The PyNEST implementation is used to initialize the connectome and neuron state variables of the microcircuit as configuration for our cluster. Furthermore, NEST simulations using the very same initializations are run on a traditional HPC cluster, serving as a golden reference to compare and verify the FPGA cluster results to. We run all simulations for 15 min of biological time.

## 3. Results

In this chapter, in accordance with the three pillars presented, we first establish a network topology and routing scheme, based on assessments with the static simulator. We then characterize the hardware system resulting from this interconnect solution to get an understanding of the system behavior. However, the dynamic simulator consists of individual components, each modeling a specific hardware unit that interacts with others, causing interfering latencies. Therefore, it is important to extract isolated information about their behavior to calibrate the dynamic simulator. For this purpose, we systematically design neural networks which first individually stress the different components of a single node and later reveal the influence of the interconnect. Finally, the dynamic simulator is calibrated using the measurements and compared to the system's real performance on the cortical microcircuit. The overarching purpose of this analysis is to (1) showcase our methodology, (2) analyze the speed and efficiency of our hardware cluster, and (3) tune the dynamic simulator so that it can reliably estimate changing system requirements posed by new neuroscience insights and applications in the future (such as changing average firing rates or more complex neuron models). This last point is key in overcoming the chicken-and-egg problem of neuromorphic simulator design described before.

**Figure 5:**
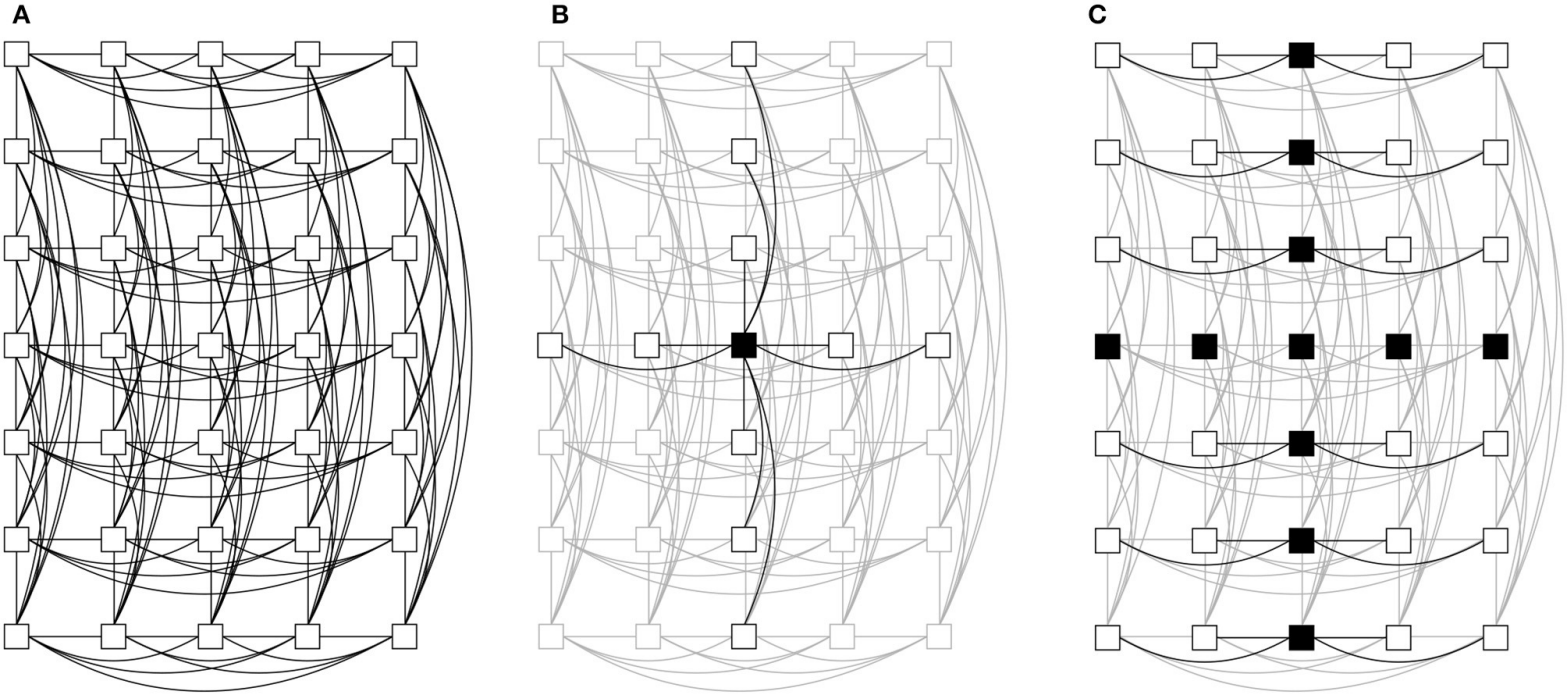
Wiring and broadcasting of messages in our cluster. **(A)** Every node is connected to all nodes on the same *x*- and *y*-axis. **(B)** Stage 1 of broadcast: a message is sent to all connected nodes. **(C)** Stage 2 of broadcast: every node with the same *x*-coordinate (except the source node) forwards the message to all other nodes of the same *y*-coordinate.

We start this analysis by devising a baseline network topology and corresponding routing algorithm using the static simulator. In previous work, we found that long-hop connections are crucial for accelerated, large-scale neuromorphic systems that contain thousands of nodes (Kauth et al., 2020). However, for our case of 35 nodes, we have enough transceivers to realize a more closely-connected topology that is simple to route through. While an all-to-all connection would require too many transceivers, a simple trade-off is the topology shown in Figure 5A. The nodes, arranged in a homogeneous 2D mesh, are each connected to all nodes in the same row (using 6.25Gbit/s SFP links) and column (using 6Gbit/s SATA links). Spikes are broadcasted in a two-step fashion—firstly, sent toward all nodes on the same *x* and *y* axes as the source node (Figure 5B), and secondly, vertically (or horizontally) forwarded from the nodes on the *x* (or *y*) axis (Figure 5C). This *xy routing* algorithm restricts the possible turns, however deadlocks cannot be eliminated (see Section 2.3.2.1). The static simulator estimates the maximum network bandwidth requirement using this topology to be < 1 Gbps/node. This is suitable and therefore, the proposed topology and routing algorithm will be used in the following.

In all following experiments, we measured the duration of the simulation τ using a driver for the FPGA cluster running on a host computer. The acceleration factor *a* is calculated based on the time resolution *h*, which is set to 0.1ms in all simulations, and the number of simulated time steps *n* according to Equation 1.


(1) $$\begin{array}{l} a=\frac{n\cdot h}{\tau } \end{array}$$

### 3.1. Characterization of nodes and interconnect

An effective way to showcase the workflow of our development framework is the exploration of limitations and bottlenecks of the compute nodes utilized in our FPGA implementation. The outcome of this characterization can be later used to both finetune the dynamic simulation and guide the development of larger compute clusters. While we will provide an analysis tailored to our compute nodes, the methodology can be generically applied to other systems.

The major bottlenecks in any distributed system can broadly be attributed to the areas of *computation, communication*, and *memory access*. Their individual influence on system performance depends on the computational load of the targeted simulation. While the computation latency is a direct function of the pipeline depth of the chosen implementation and the number of neurons calculated per parallel worker (at least for analytically solvable models), it is only expected to be a bottleneck in scenarios with low network activity. The higher the neuronal firing rates, the more synaptical information will have to be looked up. Furthermore, this can lead to major challenges in system scalability as the required communication bandwidth increases.

In this first step of characterization, we design a set of neural networks that are intended to selectively exclude usage of certain hardware components—for example, a network where no neuron fires never accesses off-chip memory. The goal is to individually explore limitations of the hardware components that are still used in these cases. While not biologically accurate, these serve to gain a better understanding of the systems bottlenecks. We implement all neuronal networks in NEST:: (Gewaltig and Diesmann, 2007) and extract information regarding neurons and synapses (neuronal states, connectome, etc.) to configure the hardware cluster. We use LIF neurons with CUBA synapses, initialized with the same model constants as neurons in the cortical microcircuit (Potjans and Diesmann, 2014). Subsequently, we obtain performance parameters such as bandwidths or latencies of the remaining components from the observed acceleration factors. These findings can be used to create simplified system models and to calibrate the dynamic simulator later on.

#### 3.1.1. Computational bottleneck

In this first test, we want to investigate the influence of computation. More precisely, we consider the time needed to compute the dynamics of a single neuron. Therefore, all kinds of inter-node communication and memory accesses should be avoided. For the realization of this test, neurons are loaded onto a single node. By setting the initial membrane potential below the action potential threshold, they are prevented from generating any spikes. However, local synchronization still starts the computation of the neuronal dynamics of the next timestep. For this purpose, each worker sends a message through the router to the scheduler after completing its computation. This influence cannot be avoided without fundamentally changing the behavior of the system. Here and in the following, the system is set up as described in Section 2.3.1.1, i.e., each node contains 10 workers that can compute up to 255 neurons each.

**Figure 6:**
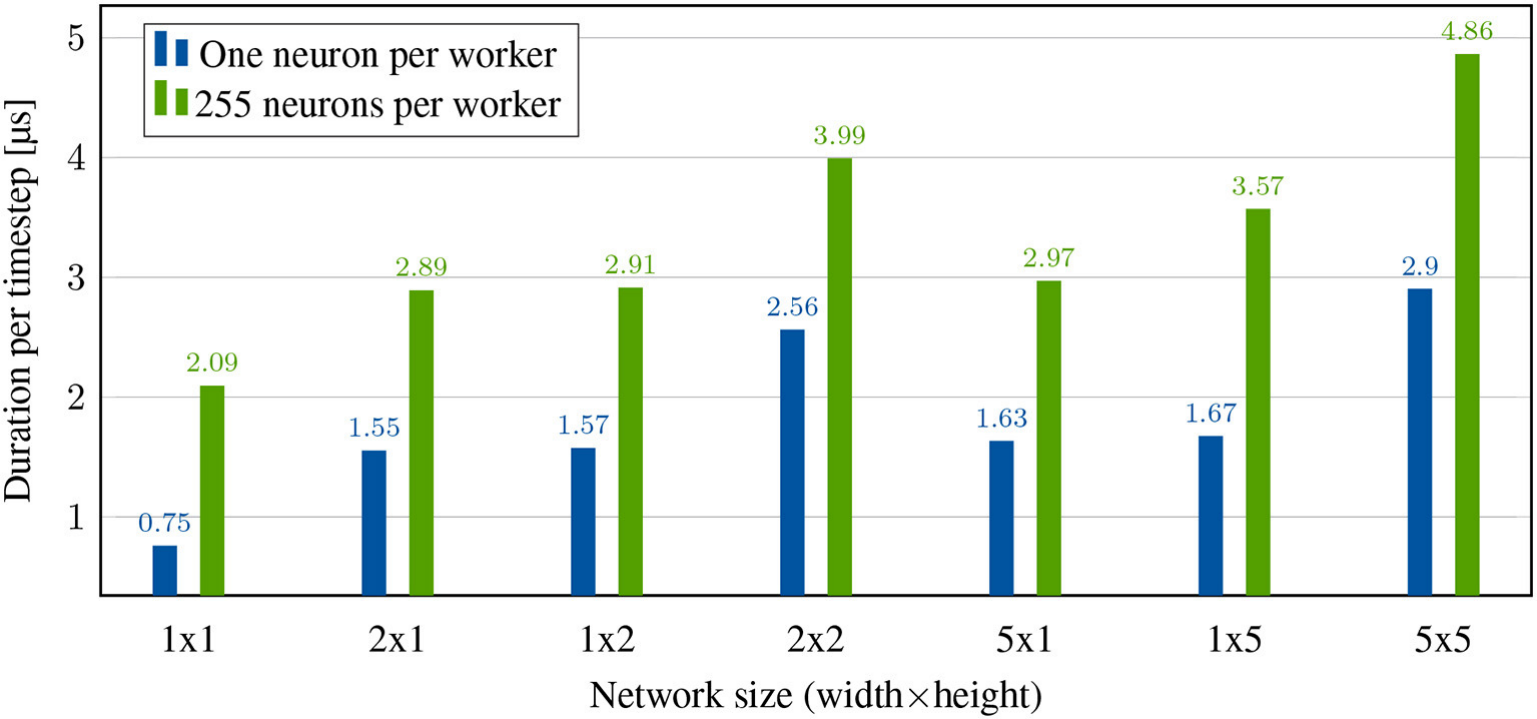
System performance without any spikes. Duration per timestep is limited by neuron computation and, depending on the cluster size, synchronization.

The bars “1 × 1” of Figure 6 show the durations per timestep of this scenario when computing different numbers of neurons per worker *NpW*. The total duration of one timestep can be expressed as τ_s_ = *t*_0_+τ_neuron_·*NpW* where τ_neuron_ is the time required to calculate a single neuron and *t*_0_ the remaining, in this case constant, duration of each timestep. The value of *t*_0_ entails all other latencies which will be investigated further in the following experiments. By means of two different values of *NpW* and τ, τ_neuron_ can be calculated by $\tau_{\text{neuron}}=\frac{\tau_{\text{s}}^{255}-\tau_{\text{s}}^{1}}{NpW^{255}-NpW^{1}}=\frac{2,090\text{ns}-755\text{ns}}{255-1}=5.256\text{ns}$. This fits the expected duration of one clock cycle of the worker operating at a frequency of 189.383MHz.

#### 3.1.2. Network latencies

In the next step, we measure transceiver latency. Again, there is no possibility for a completely isolated observation, since not all other influences can be excluded. However, the synchronization messages of our system offer the possibility to observe transceiver latency without having to generate spikes. These messages have the same size as spike messages of 128bit and, unlike spikes, do not cause memory access. We therefore deploy a neural network that, as before, never spikes. However, now it is simulated on multiple nodes instead of just one to cause synchronization messages between the nodes. Furthermore, for the sake of simplicity, only measurements with one neuron per worker will be considered below. All non-excludable influences such as local synchronization, scheduling and CDC!s (CDCs) can be eliminated by regarding the difference to the single node case.

The latency when sending a 128bit packet is: $\tau_{\text{s}}^{2\times 1}-\tau_{\text{s}}^{1\times 1}=1$,549 ns − 755ns = 794ns. This number does not represent the round-trip time because messages can be sent in both directions at the same time. However, the larger the network, the higher the chance of nodes having to wait for each other, increasing total latency. For example, the measured delay in a 5 × 1 network increases to an average of 874ns.

For the same network sizes in the y-dimension, latencies result in 815ns (1 × 2) and 944ns (1 × 5), respectively. This difference can be explained by the fact that the horizontal interconnects are operated at a frequency of 6.25Ghz instead of 6GHz and the latency of the solution is largely dependent on this. Due to the two-hop synchronization required for the present topology, we expect the duration of the largest network (5 × 5) to be approximately doubled to 1,888 ns, compared to 1 × 5. The measurement results indicate a small additional overhead with a duration of 2,144 ns, which can again be attributed to the larger network size.

#### 3.1.3. Local communication

Next, the impact of spikes on system runtime must be examined. A spike passes through multiple interacting hardware components on each node that introduce additional latencies and bandwidth limitations. This behavior has to be captured by respective modules of the dynamic simulator. For this purpose, we configure neurons to generate one action potential at each timestep. This is achieved by changing the neuronal refractory period to 0.1ms and applying the maximum possible external input current. Furthermore, we set each neuron's fanout to zero to avoid lookups, minimizing the influence of memory accesses.

**Figure 7:**
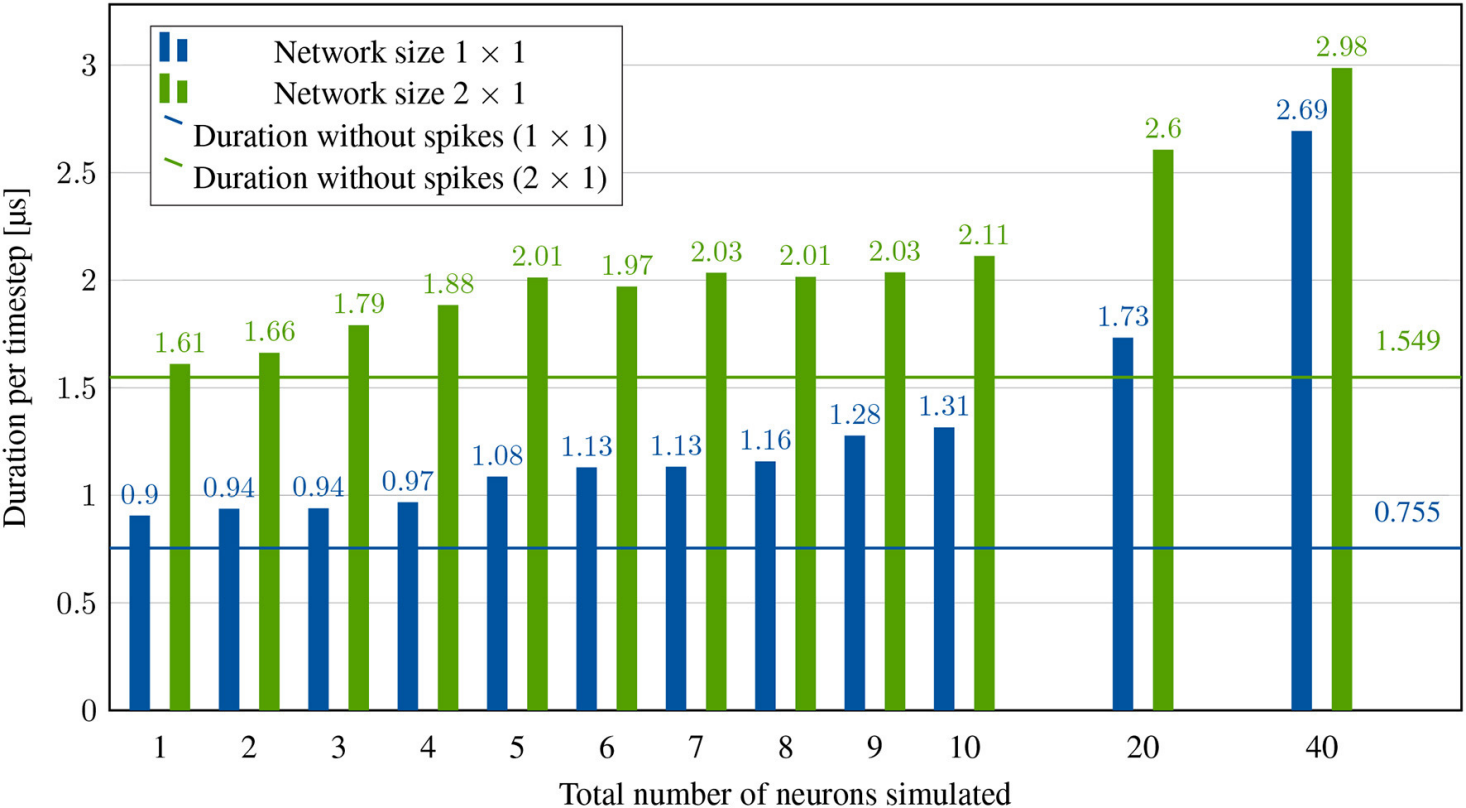
System performance with spikes, but without memory lookups. Acceleration factor is limited by neuron computation, synchronization and spike transmission.

Figure 7 shows the results of this experiment for different numbers of neurons on one and two nodes. We now investigate the overhead of a single spike generation, based on the first bar in Figure 7. Compared to the simulation of a single neuron that never spikes, a timestep now lasts 0.903ns − 0.755ns= 148ns longer. This time difference includes local routing and a single memory lookup (which is always performed by the system to retrieve the length of the synaptic list for each incoming spike). However, a large part of this delay is consumed by the local synchronization packets even without a spike, which is why this difference cannot be broken down into individual contributions. Presumably, however, the major part is due to the memory latency. Scaling up to generating 20 spikes by 20 neurons, the impact per spike is calculated to be $\frac{1.73\mu \text{s}-0.903\mu \text{s}}{20-1}=43.526\text{ns}$.

On two nodes, generating a single spike takes only 59ns longer than simulating without spikes, compared to the 148ns overhead on one node. This is expected given that some of the processes involved in the spike handling can take place in parallel to the synchronization between the nodes. Calculating the effects of a spike with 20 neurons per node yields $\frac{2.984\mu \text{s}-1.608\mu \text{s}}{40/2-1}=72.421\text{ns}$. This is significantly larger than the impact per spike on one node because we use broadcasting. With two nodes, each node has to process locally generated spikes *and* incoming spikes from other nodes.

#### 3.1.4. Memory bandwidth

Now the memory connection, which has been largely ignored up to now, will be measured to calibrate the simulator's memory model. Here, again, we will try to exclude as many other influences as possible. Accordingly, the following experiments are carried out on a single node only, such that no network communication takes place. Furthermore, to avoid congestion after the memory lookup, the target neurons of the synaptic lists are assigned in a round-robin fashion and therefore evenly distributed over the workers.

In principle, memory accesses can be characterized by two main factors: access latency and data transfer rate. To be able to determine these two separately, different measurements have to be carried out. We decided to vary both the number of accesses and the amount of data requested per access. To measure the resulting memory bandwidth, we count the number of memory accesses during one simulation. Then, we divide the number of bytes read from memory during the entire simulation by the simulation's runtime. This gives us an average memory bandwidth for one run. However, the existing overheads for the computation of neuron dynamics as well as the already described local synchronization are part of these measurement results.

**Figure 8:**
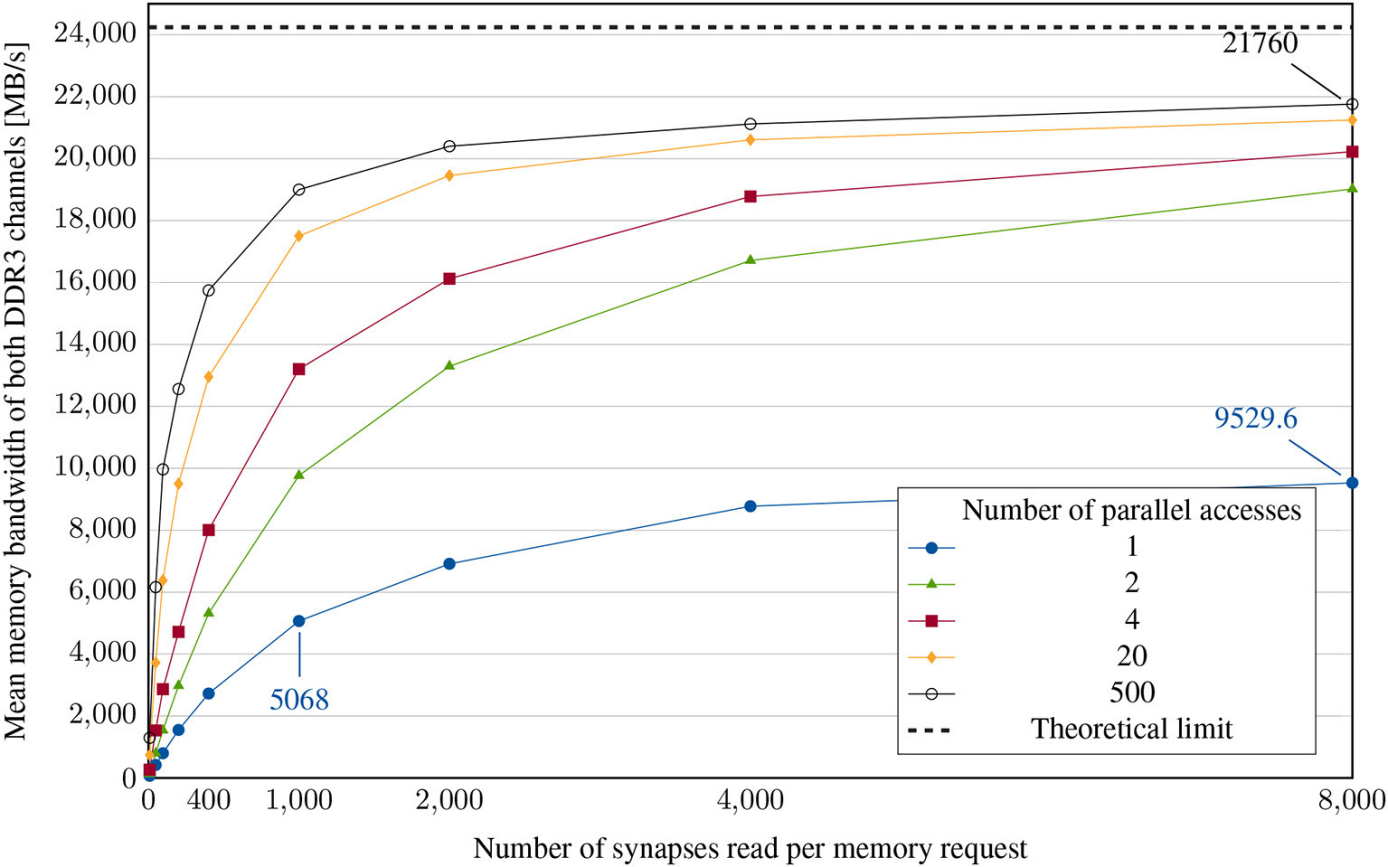
Memory bandwidth experiment: achieved mean memory bandwidth for different numbers of parallel accesses and different synaptic list lengths. The neural network is configured to have evenly distributed synapses and therefore less congestion in subsequent components of the system, enabling this case to examine the lookup bottleneck.

To conduct the experiments, each neuron is again configured to generate an action potential in each timestep so that timesteps without a lookup do not have to be accounted for in the calculation. Our first variation parameter, the number of accesses, can be varied by changing the number of neurons - each additional neuron creates an additional parallel memory read request (e.g., four neurons result in four parallel requests). The amount of requested data per access is set by the neuronal fanout. The results of sweeping both parallel accesses and request lengths is shown in Figure 8.

As expected, the average memory bandwidth utilization increases with both the requested list length and the number of requests. A single request can only use one of the two memory channels and therefore results in less than half of the maximum achievable data transfer rate. The experiment shows that in the optimal case, the hardware achieves at least 90% of the theoretically possible bandwidth for long synaptic lists. Consequently, there is hardly any further potential for optimization in this case, given that the memory is not utilized at all times. With shorter synaptic lists, however, the achieved bandwidth drops drastically and can only be compensated to a limited extent by the parallelization of requests. For significantly larger clusters, a lookup in the target node, as performed in the case of broadcast, becomes difficult. Possible solutions to this problem have already been presented by Kauth et al. (2020).


(2) $$\begin{array}{l} t_{\text{mem}}=t_{\text{lat}}+\frac{n_{\text{bytes}}}{BW_{\text{max}}}=\frac{n_{\text{bytes}}}{BW_{\text{mean}}} \end{array}$$

Equation 2 shows the relationship between memory latency, bandwidth and access time. In general, a certain time *t*_lat_ elapses between the memory request and the first byte received. In the case of DDR-SDRAM, this is often one to several 100ns. In a simplified model, the requested data is then transferred with the available maximum memory bandwidth *BW*_max_. In our measurements, besides the number of bytes requested, only the total access time is available. Figure 8 shows the apparent average memory bandwidth *BW*_mean_. Based on the measured data, the approximate memory latency is calculated using Equation 2 as *t*_lat_ = 1,000$\text{Synapses}\cdot 8\frac{\text{Byte}}{\text{Synapse}}\cdot \left(\frac{1}{5\text{GB/s}}-\frac{1}{10\text{GB/s}}\right)=800\text{ns}$. This value is far above the real memory latency. It includes several other latencies occuring during spike generation and lookup. Nevertheless, it can be used for calibration of the dynamic simulator.

#### 3.1.5. Local routing and ring buffer

**Figure 9:**
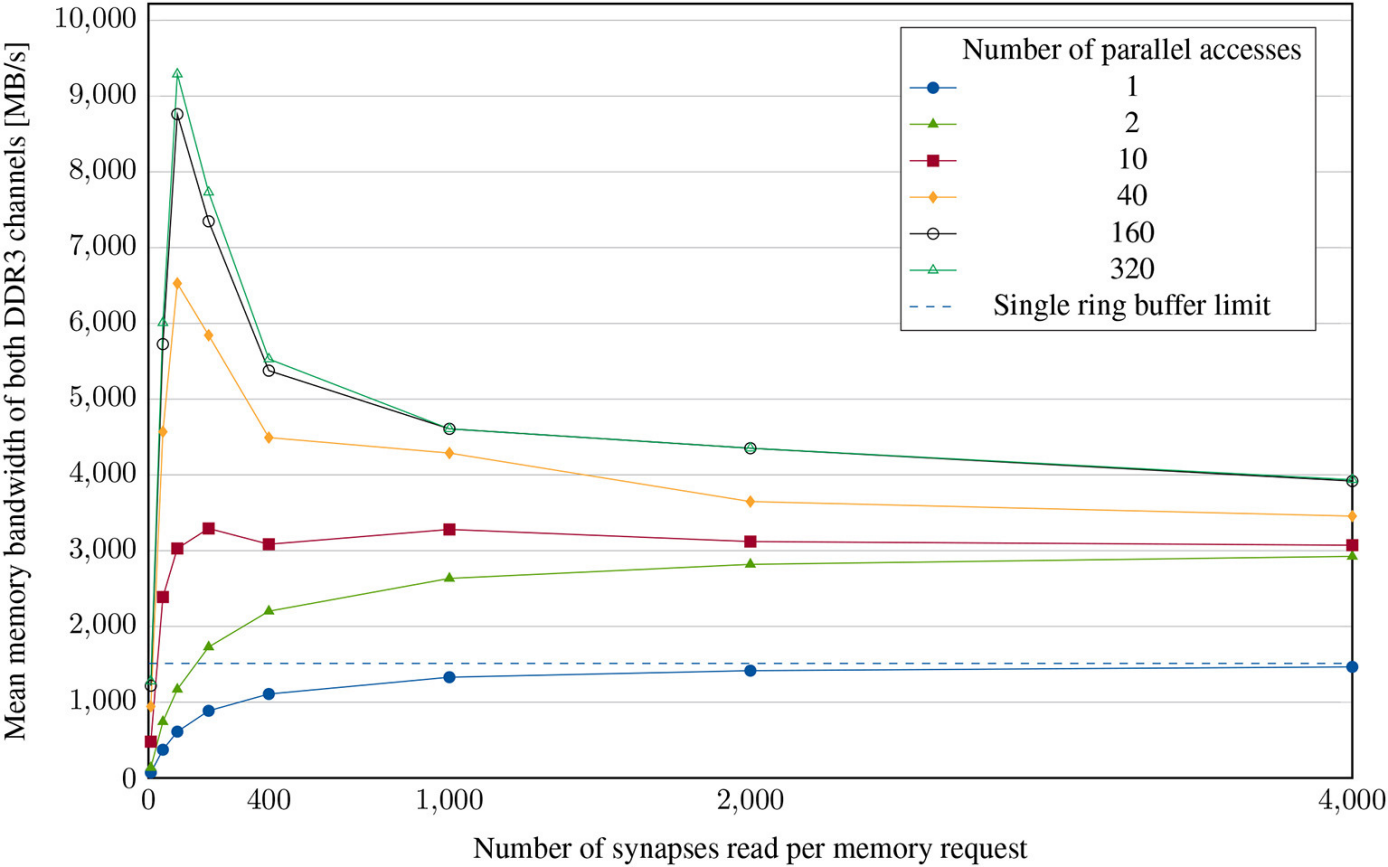
Memory bandwidth experiment: achieved mean memory bandwidth for different numbers of parallel accesses and different synaptic list lengths. The neural network is configured to have unevenly distributed synapses and therefore more congestion in subsequent components of the system, enabling this case to examine the ring buffer bottleneck.

In the previous experiment, we assumed that the targets of the synaptic lists read from memory are evenly distributed to explore the memory's capabilities. In practice, however, this may not always be the case. Local routing, consisting of a simple round-robin arbitration, can become a bottleneck if many spikes target the same ring buffer. Consequently, if the targets of synaptic lists are unevenly distributed to a significant extent, the data transfer rate of the lookup is limited due to back-pressure. In the following experiment, we will therefore determine the achievable total bandwidth of the ring buffer for such poorly distributed synaptic targets. For this purpose, the synaptic lists are generated in such a way that each neuron has only synaptic connections to itself (multiple autapses). As shown in Figure 9, the synaptic list length and the number of neurons, and thus the number of parallel requests, are varied, just like in the previous experiment.

Now, for a single access, the bandwidth converges as expected to the bandwidth of a single ring buffer of ~1.5GB/s. Similarly, the total bandwidth of two parallel accesses converges to about 3GB/s. For more than two simultaneous accesses, an anomaly can be observed. The total bandwidth increases rapidly to a list length of 96 synapses, only to drop again significantly. While the increase can be explained by the higher speed of the DRAM when fetching longer lists, as already demonstrated in the previous experiment, the subsequent drop is due to the poor distribution of synaptic targets. With small synaptic list lengths, frequent switching of the local router takes place. Internal FIFOs can thus compensate for the limitation of individual ring buffers. However, this becomes increasingly difficult in the case of longer synaptic lists due to relatively small FIFOs.

The case demonstrated here is a worst-case scenario and realistic neural networks have lower requirements. However, depending on the degree of non-uniformity of the synaptic lists, their length and the memory bandwidth, the achieved memory bandwidth can still be significantly lowered by congestion (compared to optimal case in Figure 8). To tackle this, for example, the capacity of FIFOs prior to the ring buffers can be adjusted. It is also possible, at the cost of higher latencies, to divide the lookup of longer lists into several smaller memory requests and interlace them. However, in the upcoming experiments we will focus on the microcircuit model where the capacity of existing FIFOs were designed to be sufficient to compensate any congestion.

### 3.2. Characterization of cluster

Now that we have all the data we need to calibrate the dynamic simulator, we are interested in some other aspects of our system. Scalability is a fundamental requirement for neuromorphic simulators that want to study significant parts of the human brain. As neuroscience experiments cover widely varying degrees of network size and complexity, understanding how both small and large workloads perform on different cluster sizes is crucial. Our system serves as a validation and calibration basis for the dynamic simulator, and is therefore not designed for scalability by several factors. However, given that the hardware platform can already perform neuroscience simulations, we want to investigate which network configurations best support which complexity. The characterization of individual nodes has already shown how the additional latency and synchronization imposed by a small cluster of independent nodes counteract the decreased nodal load of off-chip memory accesses and compute. To further assess the behavior of the system, we therefore perform *strong* and *weak* scaling experiments.

In an effort to explore complex interactions between different bottlenecks, we choose the cortical microcircuit as a realistic benchmark (cf. Section 2.4). The example PyNEST implementation lends itself as a suitable testcase as it allows scaling the number of neurons with automatic adaptation of the connectivity to more or less maintain the mean firing rate.

#### 3.2.1. Strong scaling

**Figure 10:**
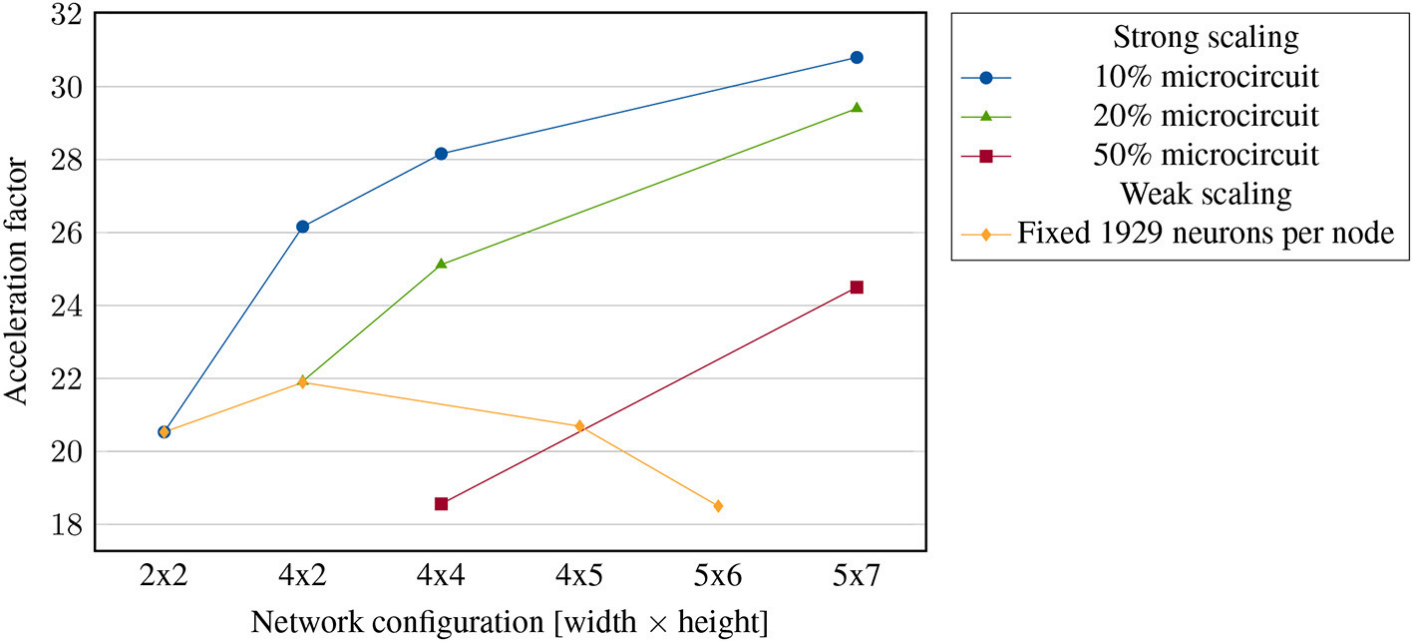
Strong and weak scaling experiment: achieved acceleration factor of the simulation of fractions of the cortical microcircuit. First, with different scalings of respectively constant total problem size, last one with a fixed problem size per node.

In strong scaling benchmarks, the number of nodes is increased while the problem size is fixed. If the required time for solving the problem reduces linearly with increasing processing power, the system is considered to show strong scaling behavior. Since the simulation of biological neural networks is largely based on communication between a large number of neurons, we don't expect strong scaling to apply here. On the contrary, in some cases, we could even expect smaller clusters of highly utilized nodes to achieve higher simulation speed, since this keeps a large part of the communication within the nodes. The optimal cluster size for computing a given neural network is a trade-off between latencies introduced by the interconnect and local bottlenecks, such as memory and computation, making it difficult to predict. Three different strong scaling experiments are shown in Figure 10. In these examples, the speed of the simulation increases with increasing cluster size, but significantly sub-linear, so that strong scaling, in the strict sense, does not hold here.

#### 3.2.2. Weak scaling

On the other hand, in weak scaling benchmarks, larger problems should be solved in the same time by using more hardware. This is a characteristic that we strictly demand from simulators of biological neuronal networks. Specifically, the cortical microcircuit with its size of about 1mm^2^ represents only a tiny part of the whole brain. Thus, to meet our long-term goal of simulating a significant portion of the human brain in an accelerated manner, a benchmark of the microcircuit is only sufficient together with the property of weak scalability.

To investigate weak scalability in our system, we performed an experiment with multiple simulations on different cluster sizes, each with 1,929 neurons per node, as shown in Figure 10. This puts our system at a relatively high load which should represent a realistic case. Network sizes below 2 × 2 were excluded since they require less than two synchronizations and are therefore not directly comparable. In general, investigating weak scaling requires a large number of nodes since small decays of operation speed can either continue or saturate with growing network sizes, e.g., when caused only by small deviations between the nodes. As can be seen, while there is a slightly decreasing trend from 4 × 2 to 5 × 6, the acceleration factors in general all aggregate around 20 × acceleration. Larger clusters are required to properly judge whether weak scaling applies or not. Previous simulations have shown, however, that the use of broadcasting prohibits scalability to a large extend (Kauth et al., 2020). Firstly, network load increases proportional to the number of nodes, independent of the neuronal fanout. Secondly, broadcasting requires postsynaptic lookups, resulting in a higher number of smaller memory accesses compared to unicasting. As shown in our previous work, long-hop-based topologies with a dedicated, directed casting scheme are suitable solutions for large scale networks. In a network of our current size, however, this scheme would still be disadvantageous.

### 3.3. Testcase: microcircuit

**Figure 11:**
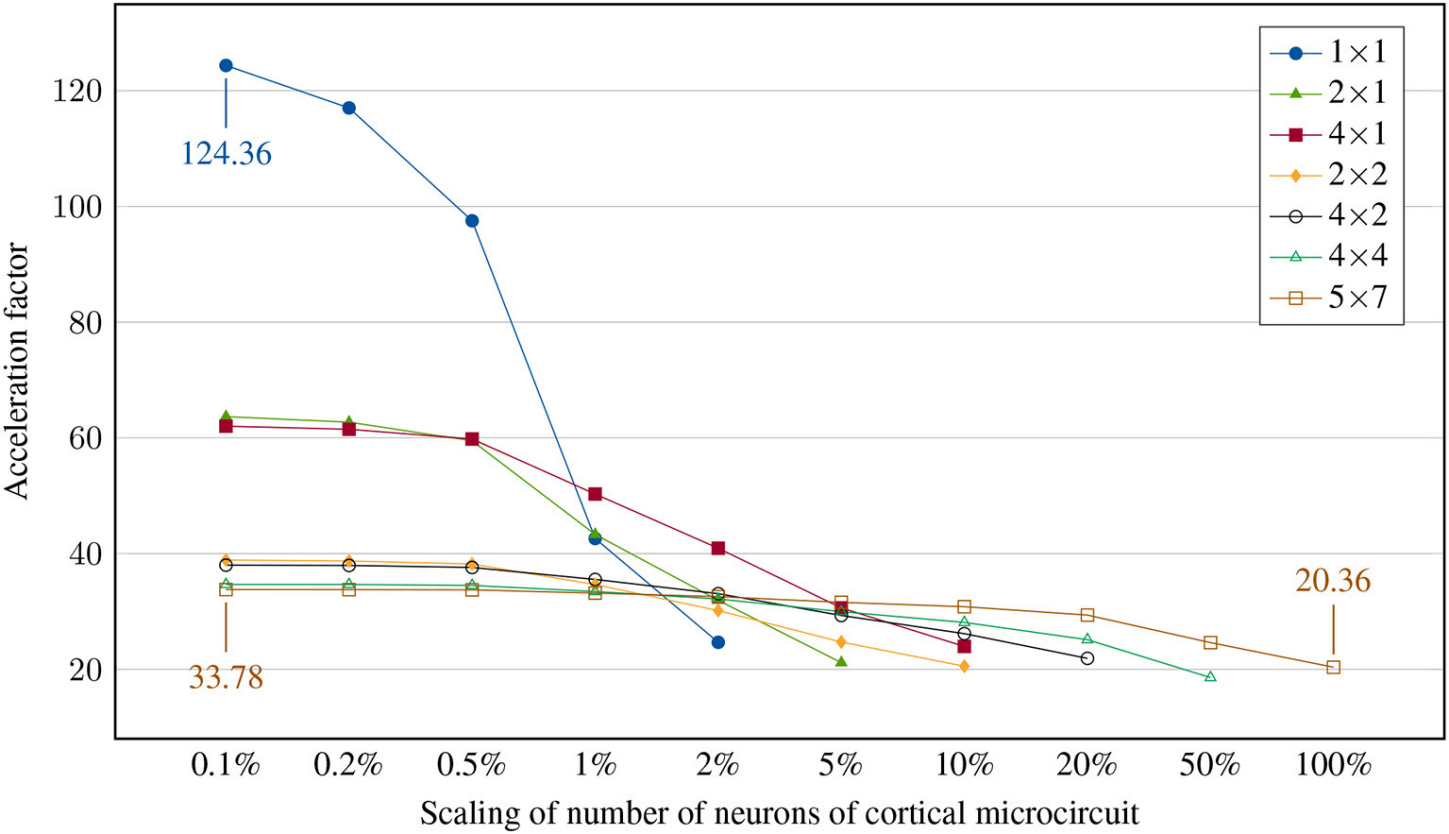
Achieved acceleration factor for cortical microcircuit at different scalings of neurons. Scaling of synapses is kept at 100%. Note that the 1 × 1 network has network synchronization disabled while 2 × 1 and 4 × 1 networks perform only a single synchronization per timestep. All other networks require two synchronizations per timestep.

To conclude the measurements on the hardware system, we perform a full sweep over several orders of magnitude by scaling the number of neurons in the cortical microcircuit model (fanout is kept at full-scale) and simulating it on different network configurations. This should answer the question how our system performs for realistic use cases of varying complexity. While previous experiments can be understood as ways to better understand the limitations and behavior of our platform, this experiment is relevant to neuroscientists who aim to accelerate and parallelize their experiments - the faster the FPGA cluster is at small- and large-scale experiments, the more usable it is to aid neuroscience research in the future. The results are shown in Figure 11.

The simulation of small neuronal networks works best on small clusters, particularly in the case of a single node where no external communication takes place, resulting in the highest achievable acceleration factor for a 0.1% microcircuit of 124.36. At larger scales, memory access starts to limit system speed. At this point, distribution among several nodes becomes advantageous. In contrast, large cluster configurations do not achieve significantly larger acceleration factors even with smallest neural networks, since synchronizations are a major limiting factor. Accordingly, scaling up the neural network reduces the simulation speed only slightly. For example, the cluster of 35 nodes reaches an acceleration factor of 33.78 when simulating 77 neurons, while the ~1,000 times larger full-scale microcircuit with 77,169 neurons can still be simulated with 20.36 ×.

### 3.4. Correctness

The entire system was designed with reproducibility and determinism as key features in mind. However, the exact simulation results will still deviate from any ground truth generated on a different system due to certain design decisions in hardware. In our case, we for example use 32bit fixed-point for saving and accumulating weights in the ring buffers before calculating the neural state update in 32bit floating-point. The resulting deviations compared to a 64bit floating-point operation are small, yet accumulate over time, possibly leading to a neuron spiking one timestep earlier or later, which in turn affects many other neurons. As spiking neural networks are chaotic systems sensitive to even small perturbations (van Vreeswijk and Sompolinsky, 1998), the resulting network activity on different systems can hardly be compared on a spike-by-spike basis.

The most simple and direct comparison to NEST can be drawn by regarding the total number of generated spikes. For a specific microcircuit initialization, 222,545,972 spikes were generated on the hardware platform, compared to 221,532,831 spikes generated when running NEST on a HPC platform. The resulting deviation of 0.46% is noticeably smaller than the difference between two different microcircuit initializations executed on the same system, which we observed to reach more than 1%. However, this comparison is fairly limited as it does not capture any dynamic behavior of the network.

To properly judge the correctness of a given simulation, we follow the established way of comparing the network activity of simulations on our system to some ground truth results, using spike-based statistics. In our case, we take NEST simulations from a high-performance computing cluster as ground truth. In particular, we compare the following well-established statistics (Gutzen et al., 2018):

- Time-average firing rates of single neurons.
- Coefficients of variation of inter-spike intervals.
- Pearson correlation coefficients between the spike trains of a randomly sampled set of 200 neurons, binned at 2ms.

**Figure 12:**
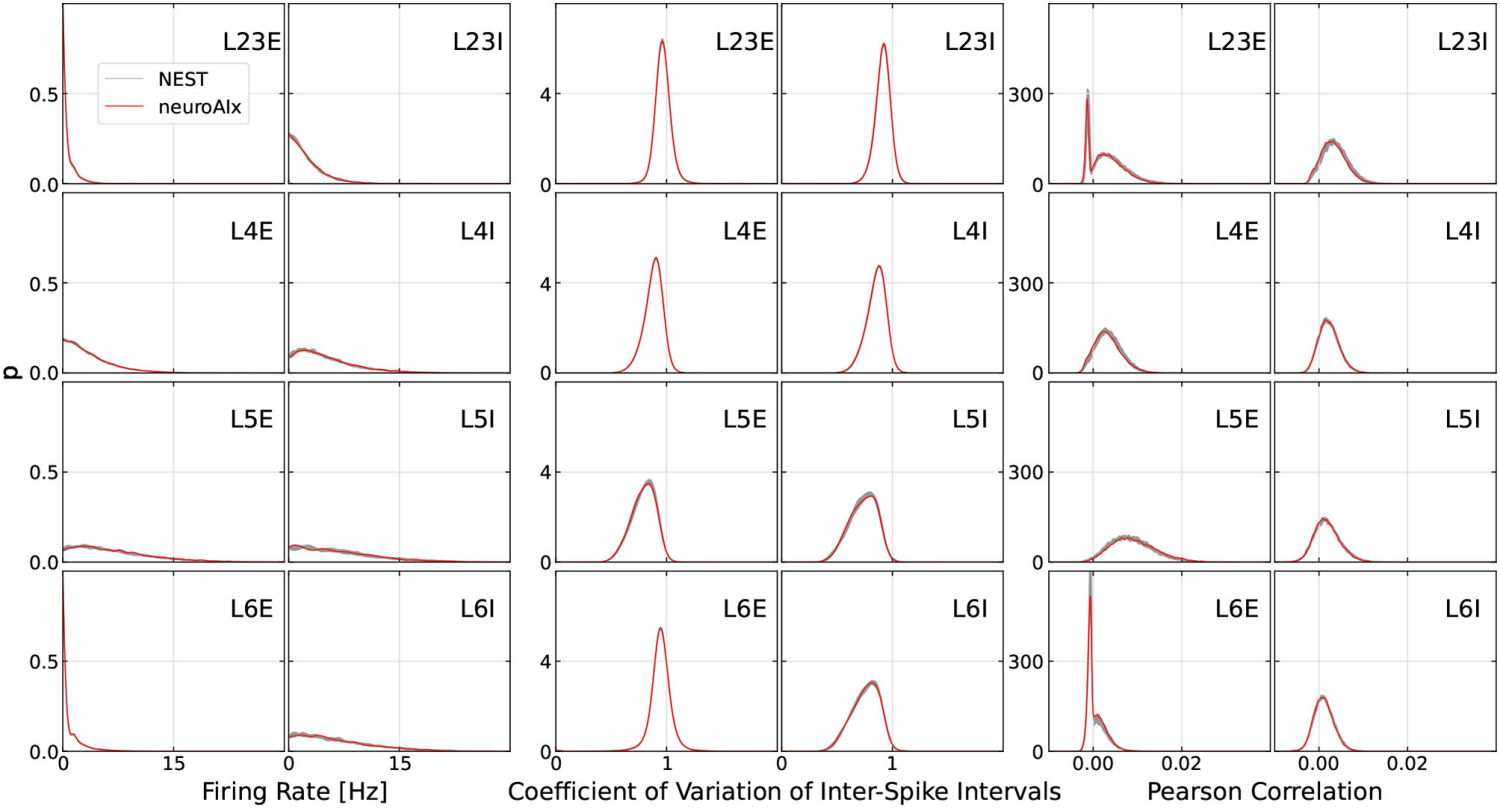
Comparison of spike-based statistics measured on our platform and 10 NEST simulations run with different seeds (min-max values marked as gray area). The simulations were run for 15 min of biological real-time.

In Figure 12, the spiking statistics of our largest experiment, the full-scale cortical microcircuit, and the corresponding results from the NEST ground truth are shown. In particular, we run 10 simulations in NEST for 15 min of biological time using different seeds to estimate the range of acceptable deviation of neuronal states and connectome (resulting min- and max values are plotted as a gray corridor). Thereby, as is common in the literature, the first 10,000 timesteps are ignored in order to exclude transient effects. We can see that the deviation of results on our cluster to the reference is minimal. Compared to second order statistics reported in other works (van Albada et al., 2018; Rhodes et al., 2019; Heittmann et al., 2022), it can be seen that we are well in the accepted range of deviation to the baseline. Neuroscience research can therefore be safely conducted on the cluster as on any other system.

### 3.5. State of the art

Before contextualizing our work within current state-of-the-art systems, we want to highlight energy efficiency as an additional metric for comparison. While not focus of the development effort of this platform, energy efficiency is generally one key motivation for developing brain-inspired algorithms and hardware. In particular, the energy per synaptic event in the human brain has been estimated to be in the range of 19–760 fJ (van Albada et al., 2018). In light of these impressive values, the neuromorphic computing community takes the human brain as a major inspiration for novel computing architectures. To better assess how close or far we are to these numbers, and to better compare to existing systems, we derived the energy consumption per synaptic event for our platform.

While running the full cortical microcircuit simulation, we measured the power consumptions of multiple nodes using a current clamp, averaged them, and verified these results inquiring the on-board power management unit via IIC. Extrapolating the resulting 26.54W to all 35 nodes, we arrive at a total power consumption of *P* = 928.9W. Using the on-board power management unit, we measured that less than half of this is consumed by the FPGA itself—off-chip memories and periphery draw most power. The resulting energy per synaptic event is calculated, as usual, as the total energy consumption of the system during simulation (given as measured power integrated over simulation time Δ*t*) divided by the number of all occurring spikes *S* times the average neuronal fanout *f*_o_, resulting in the expression: $E_{\text{syn.ev.}}=\frac{P\cdot \Delta t}{S\cdot f_{\text{o}}}$. With a total number of *S* = 222,545,972 occurring spikes, an average fanout of *f*_*o*_ = 3,880 and a simulation time of Δ*t* = 15 min/20.36, we arrive at an energy of 47.55nJ per synaptic event.

Table 1 shows the achieved acceleration and energy efficiency of various recent state-of-the-art systems. We focus this comparison on systems running the cortical microcircuit model. It has been seen in the past that efficiency measurements compare poorly when switching simulation benchmarks. For instance, while Stromatias et al. (2013) reported SpiNNaker to have an energy consumption of ~20nJ per synaptic event with a network of 200k randomly-connected Izhikevich neurons, the simulation of the cortical microcircuit drew on average 5,800 nJ per synaptic event on the same system (van Albada et al., 2018)—a difference of over two magnitudes. For this reason, comparisons to platforms not simulating the same task is inconclusive. We focus our analysis on the microcircuit due to its widespread adoption in the community as a benchmark for neuroscience simulations. As can be seen, our system compares favorably in both measures to existing platforms. In terms of speed-up, we outperform the currently fastest platform by more than 5 ×. Along the same line, our platform provides 10 × lower energy per synaptic event than the state of the art.

**Table 1:** State-of-the-art of accelerated cortical microcircuit simulation.

**Simulator**	**Hardware**	**Technology [nm]**	**Speed-up**	**Energy/Syn.Ev. [uJ]**
**This work**	**35 NetFPGAs**	**28**	**20.36**	**0.048**
CsNN [1]	305 Xilinx Zynq-7000 SoCs	28	4.06	0.783^*^
NEST [2]	2 AMD EPYC Rome	14	1.88	0.48
GeNN [3]	1 Nvidia Titan RTX	12	1.42	–
SpiNNaker [4]	318 ASICs (18 × ARM9 each)	130	1.00	0.6
NeuronGPU [5]	1 Nvidia RTX 2080 Ti	12	0.95	0.18^**^
GeNN [6]	1 Nvidia Tesla V100	12	0.54	0.47
NEST [7]	64 Intel Xeon E5-2680v3	32	0.22	5.8
SpiNNaker [7]	217 ASICs (18 × ARM9 each)	130	0.05	5.9
Human Brain [7]	–	–	1.00	19 × 10^−9^ to 760 × 10^−9^

References: [1]: Heittmann et al. (2022); [2]: Kurth et al. (2022); [3]: Knight et al. (2021); [4]: Rhodes et al. (2019); [5]: Golosio et al. (2021); [6]: Knight and Nowotny (2018); [7]: van Albada et al. (2018). Energy efficiency of the human brain roughly estimated in van Albada et al. (2018).

^*^ Energy of [1] estimated using an assumed 10W/node, leading to *P* = 3050W.

^**^ Energy of [5] estimated using an assumed 166W (based on [6], where an Nvidia Tesla V100 consumed 2/3 of its rated power for microcircuit simulation, and given a power for Nvidia RTX 2080 Ti of 250W). The bold values indicate the table entries of our work.

The energy efficiency of 48nJ per synaptic event is mainly driven by the achieved acceleration factor. Here, as well, it is important to mention that this energy efficiency is not yet the final frontier, even on our system. The off-the-shelf FPGA we use was designed as an evaluation platform and is therefore not optimized in terms of power consumption. Even in idle state, each board requires almost the full power measured during the simulation.

Reasons for the performance can be manifold and the systems are too complex to investigate exact differences. One of the reasons is local synchronization. Some other systems use global synchronization which requires packets to travel a longer distance and pass through a central network node which potentially becomes a bottleneck. Another possible reason is the network topology. Due to our small cluster size relative to SpiNNaker or CsNN, we can easily reach a high connectivity, reducing the mean network latency. However, there are solutions to solve this problems even for large cluster sizes by using long hop connections (Kauth et al., 2020) instead of neighbor-only topologies like the hexagonal mesh of SpiNNaker. Smaller systems like single GPU simulators on the other side suffer from limitations of the compute power. Memory integration also varies greatly between our system and others. General purpose computers usually have an inherently good memory interface, which is often surpassed many times over by GPUs. With these systems, the bottlenecks are likely to be elsewhere, such as in the network, which in turn creates a massive impact on their scalability. Here, the freely distributable MGTs of FPGAs are the decisive advantage over GPUs. On the one hand, GPU-based simulators impress with their simplicity in commissioning and configuration, as well as with high simulation density due to their fast memory interface and the large number of execution units. However, their communication capability, which is designed for 1-to-1 transmission, makes it difficult to combine them into larger systems. FPGA boards, on the other hand, usually have more limited memory interfaces.

### 3.6. Simulator assessment

In this last step, the first iteration of our three-pillar approach will be completed. After we used the results from our synthetic measurements, presented in Section 3.1, to calibrate the dynamic simulator, its accuracy will now be assessed. For this purpose, we use the microcircuit measurements from Section 3.3 as a reference and examine the simulator's predictions for the same scenarios. It is important to note that the measurements of the microcircuit were not used in any way to calibrate the dynamic simulator further.

**Figure 13:**
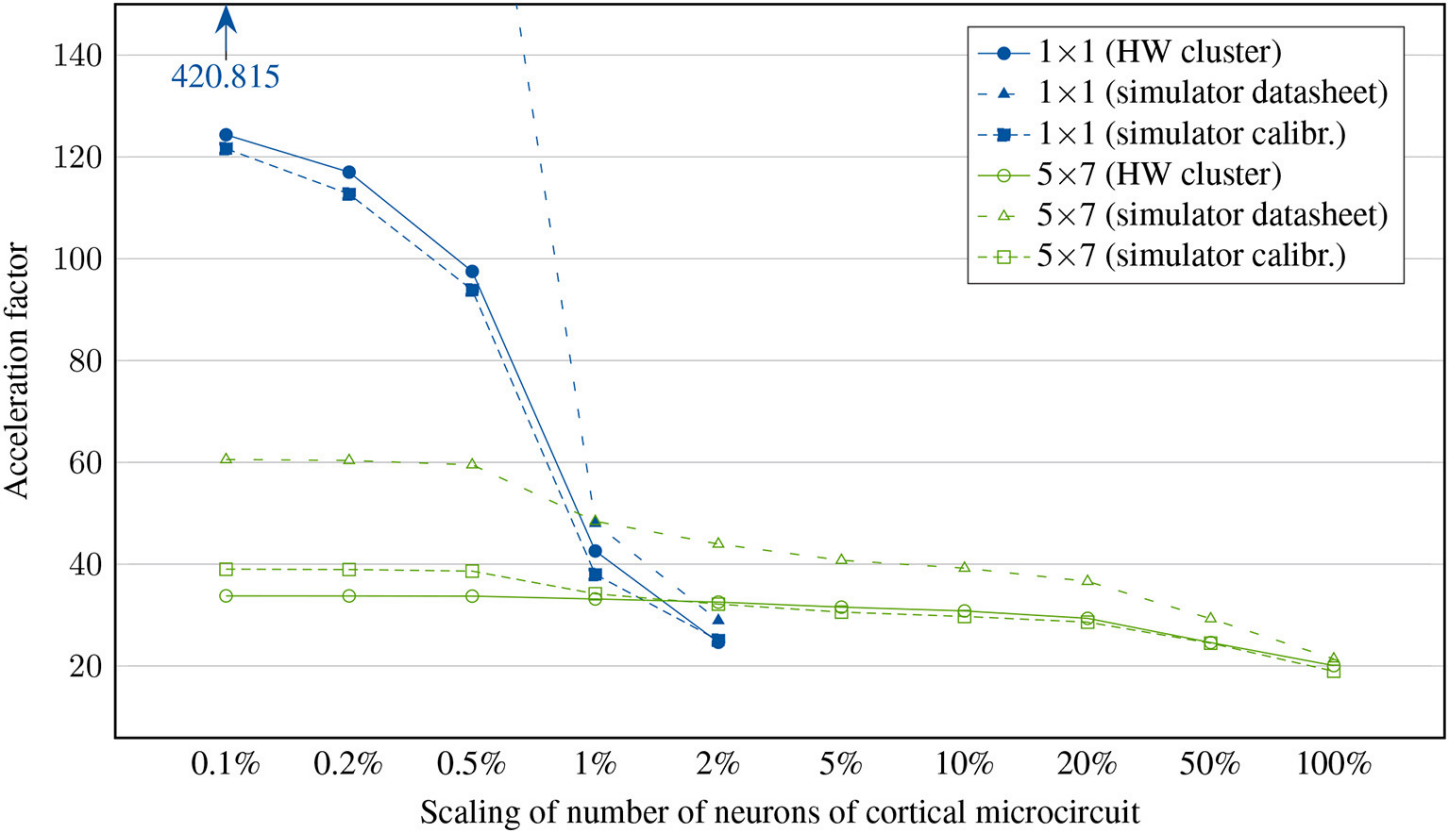
Comparison between actual measurement of the achieved acceleration factor for the cortical microcircuit on the hardware cluster against the estimation of the dynamic simulator.

Figure 13 shows the comparison between hardware measurement and the prediction of the dynamic simulator, for the smallest and largest cluster configuration and different scaling of the cortical microcircuit. While the uncalibrated simulator allows qualitative comparisons, the absolute values are far from reality. This is remarkable since the modules of the simulator were adjusted using public specifications from data sheets. After calibration, differences can still be observed, but the results now allow quantitative predictions. Furthermore, differences are to be expected, especially with small networks, since compromises were made in the development of the simulator in order to keep performance high. For example, small elements such as certain FIFOs or the multiplexing of messages were omitted, which only have a significant influence on the acceleration factor for small networks. Most importantly, the digital twin has to provide a good estimate for larger scenarios which can be confirmed by the measurements shown here.

## 4. Discussion

Our research targets the development of hardware systems that execute neuronal network models of natural density. A wide variety of solutions exists today reaching from pure software solutions running on HPC clusters over dedicated digital hardware systems to approaches mapping the computations into the analog domain. None of the existing solutions meet the future requirements on computational capability in terms of model complexity and simulation speed while, at the same time, offering the required flexibility and determinism. Flexibility is a requirement in the exploratory research as done in the domain of computational neuroscience, and determinism offers the ability to reproduce experiments and investigate noisy properties.

While being flexible, CPU or GPU based clusters are designed to support scientific simulations with fundamentally different requirements on the transformation of information, i.e., the way computations are executed and how results are communicated. Any analog and specific hardware realization of a neuromorphic system defines its capabilities during the specification phase based on the analysis of the biological requirements (e.g., average firing rates, minimal synaptic delays, etc.) that are known at the time. As much as this approach has the potential to reach high performance, it is the source of the chicken-and-egg problem as a realized system becomes outdated by the knowledge it helps to gain.

The neuroAIx-Framework overcomes these limitations by relying on three pillars: a fast analytical *static simulator* for design exploration, a slower, iterative *dynamic simulator* for accurate estimation of system behavior, and the *FPGA cluster* itself. On one side, learning in the first pillar will directly constrain the design space in the next, more involved exploration of the second pillar and so forth. On the other side, learnings, as well as calibration data, feed back to the earlier pillars to calibrate the models and thereby refine their quantitative assessment. The inaccuracy of the predictions of our cycle accurate model based on data-sheet specifications have shown the relevance for this calibration. As the models of any analyzed system architecture and neuroscience experiment are specified as code, modifications are possible throughout. More specifically, Table 2 demonstrates the flexibility of our framework by giving explicit examples of possible revisions. Depending on latest biological requirements and available hardware, the expense of adaptions of the three pillars can be estimated. In general, we consider this strictly coupled, multi-level prototyping most suitable to overcome the chicken-and-egg problem by short iteration loops due to the ability of performing rapid exploration, estimation and precise predictions.

**Table 2:** Scenario examples: required framework adjustments when changing the testcase or hardware.

**Change**	**Static simulator**	**Dynamic simulator**	**FPGA cluster**
Neuron model	No change required since neuron model is not part of the calculation	Throughput and latency parameters have to be adapted in the configuration file	For each neuron model following the same interface (lumped synapse input, spike output), the HLS implementation (in C++) of the neuron model has to be adapted, synthesis of the complete design has to be executed
Network topology	Topology can be defined in configuration file	Topology can be defined in configuration file	Cables can be connected arbitrarily. In-system soft processor automatically identifies targets behind each cable
Different FPGA Boards	No impact	Clock frequencies of system and transceivers, periphery like memory type (e.g., DDR3, DDR4) can be defined in configuration file	Neuronal accelerator is instantiated and configured (number of workers, neurons) as an IP block, peripherals (Aurora, memory controller) have to be ported
Patchy connections	Configuration file already supports definition of number, size and distance of patchy connectivity. For other changes, the code of the simulator has to be extended	The code of the simulator has to be extended	No change required, connectivity list contains complete network definition

As an application example of minimal complexity, the microcircuit was considered as as baseline. Study of prior art points to three potential bottlenecks in such hardware systems: (1) communication of spikes, (2) computation of neuronal dynamics, and (3) off-chip memory transactions. During the development of the evaluation platform, we already followed the presented methodology leading to the conclusion that a compute cluster with a proprietary communication fabric (Kauth et al., 2020) would be best suited to execute the emulation at an acceptable speed. Following the microcircuit model, we realized support for the LIF neuron model. The FPGA structure allows executing this with a high degree of parallelism in time and space. Rather instrumental are the many local memories providing on-chip storage for the system state of the individual neuron models, which already reduces the burden on the external memory interface. Evaluations based on the first and second pillar indicated having two memory channels directly attached to the programmable logic would match the performance of the other system components. At the same time, High-Bandwidth Memories (HBM) appeared of no additional benefit as the latency of random accesses is decisive. Performance evaluations of the realized system confirmed this prediction. This way, we avoided a transition to model-specific optimizations such as on-the-fly generation of connectivity information, preserving the option to upload predefined connectomes as well as the flexibility to accommodate plasticity.

Just as HPC clusters get continuously updated, more recent FPGA generations provide up to 8 × faster transceivers, four memory channels and more and faster logic resources. Hence, we see a persistent advantage in using such FPGA clusters retaining the demonstrated 20 × speed-up w.r.t. biological real-time, i.e. 10 × speed-up over non-FPGA platforms. This comes on top of the inherent flexibility and the deterministic operation of our system. Even the energy per synaptic event of 48nJ is 10 × less than any other platform although this was no optimization criterion during the design of the system. In conclusion, an upscaled FPGA cluster could act as an intermediate system solution before next-generation neuroscience simulation platforms become available. As a next step, we are realizing a high-level web-based interface to specify, execute and analyze neuroscience simulations on the cluster.

## Data availability statement

The raw data supporting the conclusions of this article will be made available by the authors, without undue reservation.

## Author contributions

KK and TS contributed to the development of the FPGA cluster, implementation of the microcircuit model, performed the simulation and data analysis, and wrote the paper. KK supervised the development of the static- and dynamic simulators while VS has contributed to the development of the dynamic simulator. TG was the supervisor of the whole project. All authors contributed to the article and approved the submitted version.

## References

[B1] Billaudelle S. Stradmann Y. Schreiber K. Cramer B. Baumbach A. Dold D.. (2020). “Versatile emulation of spiking neural networks on an accelerated neuromorphic substrate,” in 2020 IEEE International Symposium on Circuits and Systems (ISCAS) (Seville: IEEE), 1–5. 10.1109/ISCAS45731.2020.9180741

[B2] Brette R. Rudolph M. Carnevale T. Hines M. Beeman D. Bower J. M.. (2007). Simulation of networks of spiking neurons: a review of tools and strategies. J. Comput. Neurosci. 23, 349–398. 10.1007/s10827-007-0038-6 17629781 PMC2638500

[B3] Brunel N. (2000). Dynamics of sparsely connected networks of excitatory and inhibitory spiking neurons. J. Comput. Neurosci. 8, 183–208. 10.1023/A:1008925309027 10809012

[B4] Davies M. Srinivasa N. Lin T.-H. Chinya G. Cao Y. Choday S. H.. (2018). Loihi: a neuromorphic manycore processor with on-chip learning. IEEE Micro 38, 82–99. 10.1109/MM.2018.112130359

[B5] Davison A. P. Brüderle D. Eppler J. Kremkow J. Muller E. Pecevski D.. (2009). PyNN: a common interface for neuronal network simulators. Front. Neuroinform. 2, 11. 10.3389/neuro.11.011.2008 19194529 PMC2634533

[B6] Dey S. Dimitrov A. G. (2021). Mapping and validating a point neuron model on intel's neuromorphic hardware loihi. arXiv. [preprint]. 10.3389/fnins.2022.883360 35712458 PMC9197133

[B7] Diesmann M. (2018). ACA: Towards Multi-scale Natural-density Neuromorphic Computing. Available online at: https://www.fz-juelich.de/en/aca (accessed January 13, 2023).

[B8] Eppler J. M. Helias M. Muller E. Diesmann M. Gewaltig M.-O. (2009). PyNEST: a convenient interface to the NEST simulator. Front. Neuroinform. 2, 12. 10.3389/neuro.11.012.2008 19198667 PMC2636900

[B9] Furber S. B. Galluppi F. Temple S. Plana L. A. (2014). The spinnaker project. Proc. IEEE, 102, 652–665. 10.1109/JPROC.2014.2304638

[B10] Furber S. B. Lesterand D. R. Plana L. A. Garside J. D. Painkras E. Temple S.. (2013). Overview of the SpiNNaker system architecture. IEEE Trans. Comput. 62, 2454–2467. 10.1109/TC.2012.14236188479

[B11] Gewaltig M.-O. Diesmann M. (2007). Nest (neural simulation tool). Scholarpedia 2, 1430. 10.4249/scholarpedia.1430

[B12] Golosio B. Tiddia G. Luca C. D. Pastorelli E. Simula F. Paolucci P. S.. (2021). Fast simulations of highly-connected spiking cortical models using GPUs. Front. Comput. Neurosci. 15, 627620. 10.3389/fncom.2021.627620 33679358 PMC7925400

[B13] Gutzen R. Von Papen M. Trensch G. Quaglio P. Grün S. Denker M. (2018). Reproducible neural network simulations: statistical methods for model validation on the level of network activity data. Front. Neuroinform. 12, 90. 10.3389/fninf.2018.00090 30618696 PMC6305903

[B14] Heittmann A. Psychou G. Trensch G. Cox C. E. Wilcke W. W. Diesmann M.. (2022). Simulating the cortical microcircuit significantly faster than real time on the IBM INC-3000 neural supercomputer. Front. Neurosci. 15, 728460. 10.3389/fnins.2021.728460 35126034 PMC8811464

[B15] Izhikevich E. (2004). Which model to use for cortical spiking neurons? IEEE Trans. Neural Netw. 15, 1063–70. 10.1109/TNN.2004.832719 15484883

[B16] Izhikevich E. M. (2003). Simple model of spiking neurons. IEEE Trans. Neural Netw. 14, 1569–1572. 10.1109/TNN.2003.820440 18244602

[B17] Kauth K. Stadtmann T. Brandhofer R. Sobhani V. Gemmeke T. (2020). “Communication architecture enabling 100x accelerated simulation of biological neural networks,” in 2020 ACM/IEEE International Workshop on System Level Interconnect Prediction (SLIP) (San Diego, CA: ACM), 1–8. 10.1145/3414622.3431909

[B18] Kleijnen R. Robens M. Schiek M. van Waasen S. (2022). A network simulator for the estimation of bandwidth load and latency created by heterogeneous spiking neural networks on neuromorphic computing communication networks. J. Low Power Electron. Appl. 12, 23. 10.3390/jlpea12020023

[B19] Knight J. C. Komissarov A. Nowotny T. (2021). PyGeNN: a Python library for GPU-enhanced neural networks. Front. Neuroinform. 15, 136–142. 10.3389/fninf.2021.659005 33967731 PMC8100330

[B20] Knight J. C. Nowotny T. (2018). GPUs outperform current HPC and neuromorphic solutions in terms of speed and energy when simulating a highly-connected cortical model. Front. Neurosci. 12, 1–19. 10.3389/fnins.2018.00941 30618570 PMC6299048

[B21] Kurth A. C. Senk J. Terhorst D. Finnerty J. Diesmann M. (2022). Sub-realtime simulation of a neuronal network of natural density. Neuromorphic Comput. Eng. 2, 021001. 10.1088/2634-4386/ac55fc35447605

[B22] Kuśmierz Ł. Isomura T. Toyoizumi T. (2017). Learning with three factors: modulating Hebbian plasticity with errors. Curr. Opin. Neurobiol. 46, 170–177. 10.1016/j.conb.2017.08.020 28918313

[B23] Li K. Qiu Y. Jiang C. Malawski M. Nabrzyski J. (2020). “Improving system utilization on wireless HPC systems with torus interconnects,” in 2020 IEEE 22nd International Conference on High Performance Computing and Communications; IEEE 18th International Conference on Smart City; IEEE 6th International Conference on Data Science and Systems (HPCC/SmartCity/DSS) (Yanuca Island: IEEE), 60–69. 10.1109/HPCC-SmartCity-DSS50907.2020.00009

[B24] Mayr C. Hoeppner S. Furber S. (2019). Spinnaker 2: a 10 million core processor system for brain simulation and machine learning. arXiv. [preprint]. 10.48550/arXiv.1911.02385

[B25] Meyer M. Kenter T. Plessl C. (2022). Multi-FPGA designs and scaling of HPC challenge benchmarks via MPI and circuit-switched inter-FPGA networks. arXiv. [preprint]. 10.1145/3576200

[B26] Moore S. W. Fox P. J. Marsh S. J. Markettos A. T. Mujumdar A. (2012). “Bluehive - a field-programable custom computing machine for extreme-scale real-time neural network simulation,” in 2012 IEEE 20th International Symposium on Field-Programmable Custom Computing Machines (Toronto, ON: IEEE), 133–140. 10.1109/FCCM.2012.32

[B27] Panchapakesan S. Fang Z. Li J. (2022). Syncnn: evaluating and accelerating spiking neural networks on fpgas. ACM Trans. Reconfigurable Technol. Syst. 15, 1–27. 10.1145/3514253

[B28] Potjans T. C. Diesmann M. (2014). The cell-type specific cortical microcircuit: relating structure and activity in a full-scale spiking network model. Cereb. Cortex, 24, 785–806. 10.1093/cercor/bhs358 23203991 PMC3920768

[B29] Rhodes O. Peres L. Rowley A. G. D. Gait A. Plana L. A. Brenninkmeijer C.. (2019). Real-time cortical simulation on neuromorphic hardware. Phil. Trans. R. Soc. A 378, 20190160. 10.1098/rsta.2019.0160 31865885 PMC6939236

[B30] Rolls E. T. Deco G. (2010). The Noisy Brain: Stochastic Dynamics as a Principle of Brain Function, Vol. 34. Oxford: Oxford University Press. 10.1093/acprof:oso/9780199587865.001.0001

[B31] Roth U. Jahnke A. Klar H. (1995). “Hardware requirements for spike-processing neural networks,” in From Natural to Artificial Neural Computation, eds F. Sandoval, and J. Mira (Berlin, Heidelberg: Springer Berlin Heidelberg), 720–727. 10.1007/3-540-59497-3_243

[B32] Rotter S. Diesmann M. (1999). Exact digital simulation of time-invariant linear systems with applications to neuronal modeling. Biol. Cybern. 81, 381–402. 10.1007/s004220050570 10592015

[B33] Schemmel J. Fieres J. Meier K. (2008). “Wafer-scale integration of analog neural networks,” in 2008 IEEE International Joint Conference on Neural Networks (IEEE World Congress on Computational Intelligence) (Hong Kong: IEEE), 431–438. 10.1109/IJCNN.2008.4633828

[B34] Senk J. Hagen E. van Albada S. J. Diesmann M. (2018). Reconciliation of weak pairwise spike-train correlations and highly coherent local field potentials across space. arXiv. [preprint]. 10.48550/arXiv.1805.10235 PMC11513197 39462814

[B35] Silver R. Boahen K. Grillner S. Kopell N. Olsen K. L. (2007). Neurotech for neuroscience: unifying concepts, organizing principles, and emerging tools. J. Neurosci. 27, 11807–11819. 10.1523/JNEUROSCI.3575-07.2007 17978017 PMC3275424

[B36] Sobhani V. Kauth K. Stadtmann T. Gemmeke T. (2022). Deadlock-freedom in computational neuroscience simulators. IEEE Design Test 39, 70–78. 10.1109/MDAT.2022.3204199

[B37] Stromatias E. Galluppi F. Patterson C. Furber S. (2013). “Power analysis of large-scale, real-time neural networks on spinnaker,” in The 2013 International Joint Conference on Neural Networks (IJCNN) (Dallas, TX: IJCNN), 1–8. 10.1109/IJCNN.2013.6706927

[B38] van Albada S. J. Pronold J. van Meegen A. Diesmann M. (2020). Usage and scaling of an open-source spiking multi-area model of monkey cortex. arXiv. [preprint]. 10.1007/978-3-030-82427-3_4

[B39] van Albada S. J. Rowley A. G. Senk J. Hopkins M. Schmidt M. Stokes A. B.. (2018). Performance comparison of the digital neuromorphic hardware SpiNNaker and the neural network simulation software NEST for a full-scale cortical microcircuit model. Front. Neurosci. 12, 291. 10.3389/fnins.2018.00291 29875620 PMC5974216

[B40] van Vreeswijk C. Sompolinsky H. (1998). Chaotic balanced state in a model of cortical circuits. Neural Comput. 10, 1321–1371. 10.1162/089976698300017214 9698348

[B41] Vogels T. P. Abbott L. F. (2005). Signal propagation and logic gating in networks of integrate-and-fire neurons. J. Neurosci. 25, 10786–10795. 10.1523/JNEUROSCI.3508-05.2005 16291952 PMC6725859

[B42] Wang R. Hamilton T. Tapson J. van Schaik A. (2014). “An FPGA design framework for large-scale spiking neural networks,” in 2014 IEEE International Symposium on Circuits and Systems (ISCAS), (Melboune, VIC: IEEE), 457–460. 10.1109/ISCAS.2014.6865169

[B43] Wang R. M. Thakur C. S. Van Schaik A. (2018). An fpga-based massively parallel neuromorphic cortex simulator. Front. Neurosci. 12, 213. 10.3389/fnins.2018.00213 29692702 PMC5902707

[B44] Yavuz E. Turner J. Nowotny T. (2016). GeNN: a code generation framework for accelerated brain simulations. Sci. Rep. 6, 18854. 10.1038/srep18854 26740369 PMC4703976

[B45] Zilberman N. Audzevich Y. Covington G. A. Moore A. W. (2014). NetFPGA SUME: toward 100 Gbps as research commodity. IEEE Micro 34, 32–41. 10.1109/MM.2014.61

